# Exceptional sulfur and iron isotope enrichment in millimetre-sized, early Palaeozoic animal burrows

**DOI:** 10.1038/s41598-020-76296-8

**Published:** 2020-11-20

**Authors:** Dario Harazim, Joonas J. Virtasalo, Kathryn C. Denommee, Nicolas Thiemeyer, Yann Lahaye, Martin J. Whitehouse

**Affiliations:** 1 Department of Geosciences, University of Calgary, 2500 University Drive NW, Calgary, AB T2N 1N4 Canada; 2 Department of Geology and Geophysics, Louisiana State University, E235 Howe-Russel-Kniffen Geoscience Complex, Baton Rouge, LA 70803 USA; 3 Geological Survey of Finland (GTK), P.O. Box 96, 02151 Espoo, Finland; 4 ExxonMobil Upstream Research Company, 22777 Springwoods Village Parkway, Spring, TX 77389 USA; 5 Viscom AG, Carl-Buderus-Strasse, 6-15, 30455 Hannover, Germany; 6 Finland Isotope Geoscience Laboratory, Geological Survey of Finland (GTK), P.O. Box 96, 02151 Espoo, Finland; 7 Department of Geosciences, Swedish Museum of Natural History, Box 50007, 104 05 Stockholm, Sweden

**Keywords:** Element cycles, Element cycles

## Abstract

Pyrite-δ^34^S and -δ^56^Fe isotopes represent highly sensitive diagnostic paleoenvironmental proxies that express high variability at the bed (< 10 mm) scale that has so far defied explanation by a single formative process. This study reveals for the first time the paleoenvironmental context of exceptionally enriched pyrite-δ^34^S and -δ^56^Fe in bioturbated, storm-reworked mudstones of an early Ordovician storm-dominated delta (Tremadocian Beach Formation, Bell Island Group, Newfoundland). Very few studies provide insight into the low-temperature sulfur and iron cycling from bioturbated muddy settings for time periods prior to the evolution of deep soil horizons on land. Secondary ion mass spectroscopy (SIMS) analyses performed on Beach Formation muddy storm event beds reveal spatially distinct δ^34^S and δ^56^Fe values in: (a) tubular biogenic structures and trails (δ^34^S ~ +40‰; δ^56^Fe ~ −0.5‰), (b) silt-filled *Planolites* burrows (δ^34^S ~ +40‰; δ^56^Fe ~ +0.5 to + 2.1‰), and (c) non-bioturbated mudstone (δ^34^S ~ +35‰; δ^56^Fe ~ +0.5‰). δ^34^S values of well above + 40.0‰ indicate at least some pyrite precipitation in the presence of a ^34^S-depleted pore water sulfide reservoir, via closed system (Raleigh-type) fractionation. The preferential enrichment of ^56^Fe in *Planolites* burrows is best explained via microbially-driven liberation of Fe(II) from solid iron parent phases and precipitation from a depleted ^54^Fe dissolved Fe(II) reservoir. Rigorous sedimentological analysis represents a gateway to critically test the paleoenvironmental models describing the formation of a wide range of mudstones and elucidates the origins of variability in the global stable S and Fe isotope record.

## Introduction

**Figure 1:**
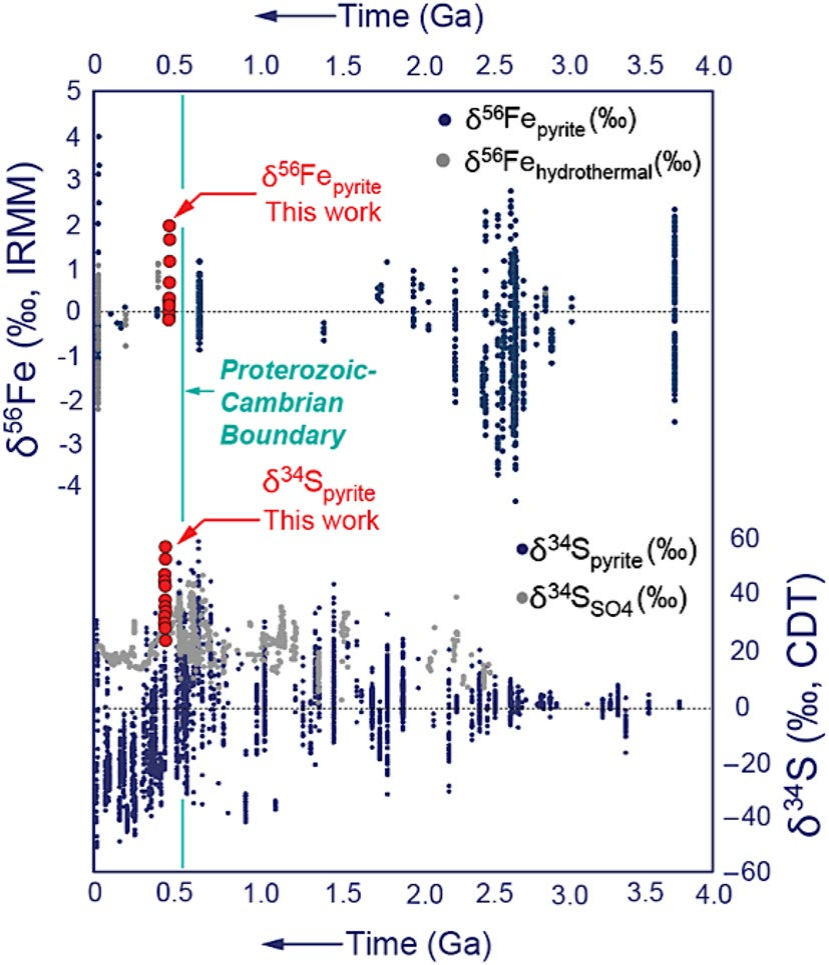
This figure shows the global compilation of δ^34^S in sedimentary pyrite and sulfate, as well as the δ^56^Fe measured from pyrite and iron-bearing hydrothermal phases versus the δ^34^S and δ^56^Fe data measured in the Lower Beach Formation. This compilation also includes other published datasets^2,7,12,52^.

Pyrite-δ^34^S and -δ^56^Fe measured from mudstones are regularly employed by earth scientists as powerful paleoenvironmental proxies that resolve the chemical composition and functioning of earth’s ocean–atmosphere reservoir through geologic time^1–7^. Pyrite-δ^34^S and -δ^56^Fe records are, however, inherently noisy and appear, in some instances, significantly enriched above parent phase isotope signatures (Fig. 1). The origin of this enrichment has so far defied explanation by a single process. As pyrite precipitates within sediment, its diagnostic stable sulphur isotope signature captures the fractionation conditions of the specific diagenetic zone in which a respective pyrite phase precipitates^8, 9^. Millimetre-sized, pyritic animal burrows feature prominently in our effort to understand and quantify the cycling of sulphur and iron in natural systems through geologic time^10–12^. Pyrite precipitation in animal burrows occurs very early, prior to sediment compaction^9,13^. The precipitating pyrite within the burrow walls represents the terminal step in the cycling of sulphur and iron^14^, thereby functioning as an important archive for early diagenetic conditions of ancient sediment pore waters^2^. In numerous modern and ancient studies, diagnostic pyrite-δ^34^S signatures have previously been shown to (a) reflect changes in the pathway of stable S isotope fractionation within the diagenetic sequence^11,15^, (b) reveal the most likely reductants driving the formation of pyrite^16^ and (c) mirror how individual dissolved sulfur reservoirs interact during diagenesis^17,18^. Despite being fractionated synchronously during the formation of pyrite, δ^56^Fe is, unfortunately, rarely measured along with δ^34^S from the same pyrite grain^12,19^. Within this study we present for the first time a paired sedimentary pyrite-δ^34^S and -δ^56^Fe record from exceptionally preserved millimetre-sized, pyritic animal burrows of an early Ordovician, fine-grained, high-energy deltaic coastline. Previous detailed textural and fabric evidence yielded that Beach Formation mudstones were initially deposited via pulsed sedimentation events, which were then repeatedly reworked by waves and currents^20,21^. The Beach Formation deposits thereby represent one of the very few, excellently preserved early Paleozoic muddy coastlines with a detailed sedimentological analysis of the mud-dominated portion^21^.

Unraveling the isotopic signature of animal-sediment interactions within excellently preserved early Paleozoic shallow-marine systems offers a unique opportunity to expand our knowledge about pathways of organic matter remineralization prior to the evolution of terrestrial soils and better understand the workings of the shallow-marine sulphur and iron cycle in a time period and a depositional system for which few such measurements exist^7^. It is therefore important to understand which microbial respiration strategies make pyrite in high-energy seafloor environments and how bed deposition, bed erosion and macrofaunal sediment colonization control the stable S- and Fe-isotope signature of diagenetic pyrite. Expanding our patchy knowledge about the functioning of ancient shallow, muddy depositional systems is critical, because even conservative estimates showcase that today’s muddy tide- and wave-dominated coastline sediments respire high volumes of riverine and marine organic matter and store ~ 60% of all generated sedimentary organic matter on our planet^22–24^.

### Sedimentologic context of the ^34^S- and ^56^Fe-enriched pyrites of the Beach Formation

**Figure 2:**
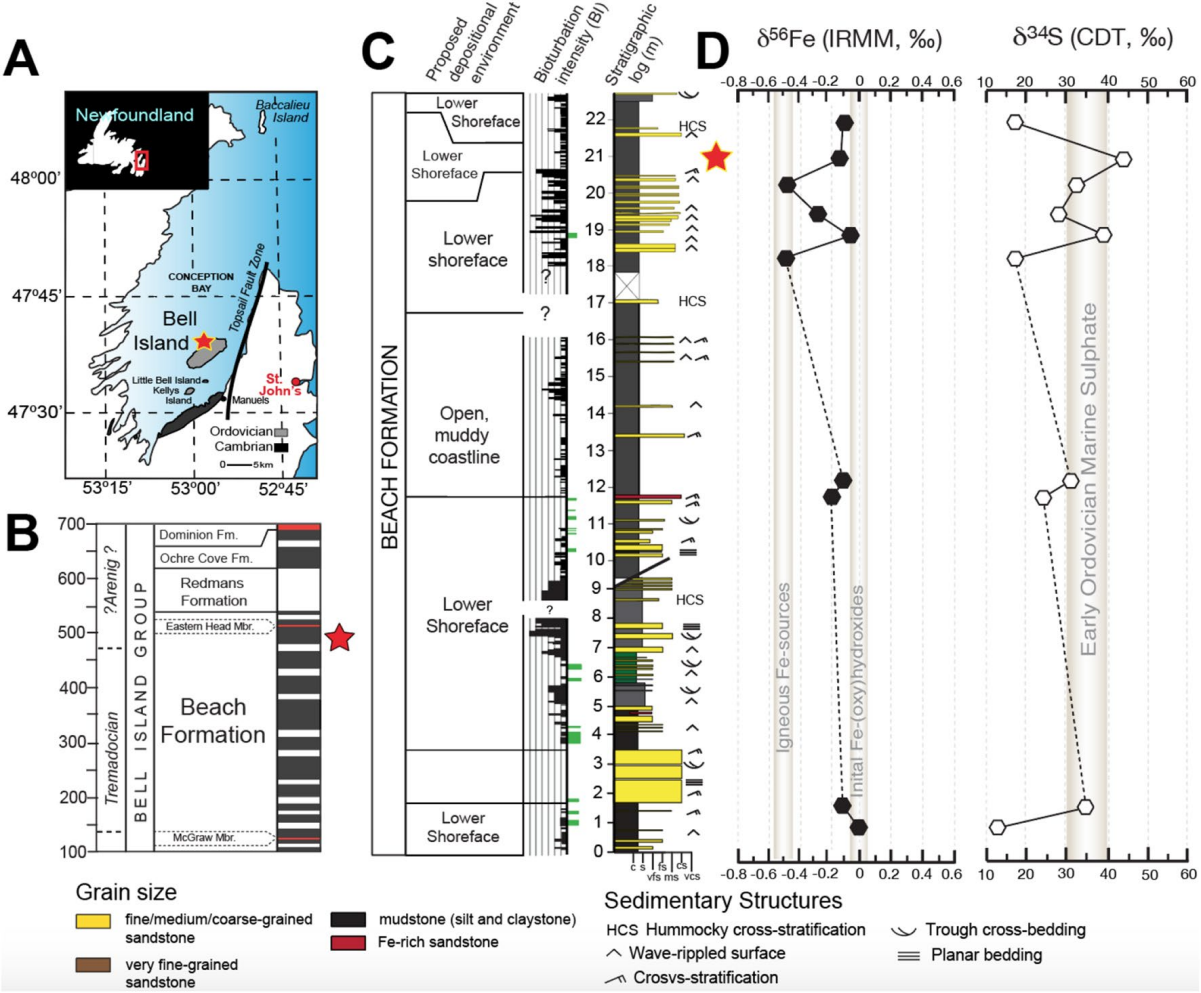
Geologic background and stable Fe and S characteristics of the Beach Formation at Freshwater Cove, Bell Island, Newfoundland. (**A**) shows the location of Bell Island as well as the distribution of Cambrian and Ordovician Strata in and around Conception Bay, Newfoundland^26^. (**B**) Simplified stratigraphy of the Early Ordovician Bell Island Group, and sampling interval (marked by red star). (**C)** Stratigraphic column of the Freshwater Cove section. The interval containing the pyritic structures analyzed in this study is marked with a red star. (**D**) shows bulk rock measurements of δ^34^S and δ^56^Fe at Freshwater Cove (Parsonville). Whole-rock organic carbon contents (TOC, wt.%) are well below 1.0 wt.%^20^.

**Figure 3:**
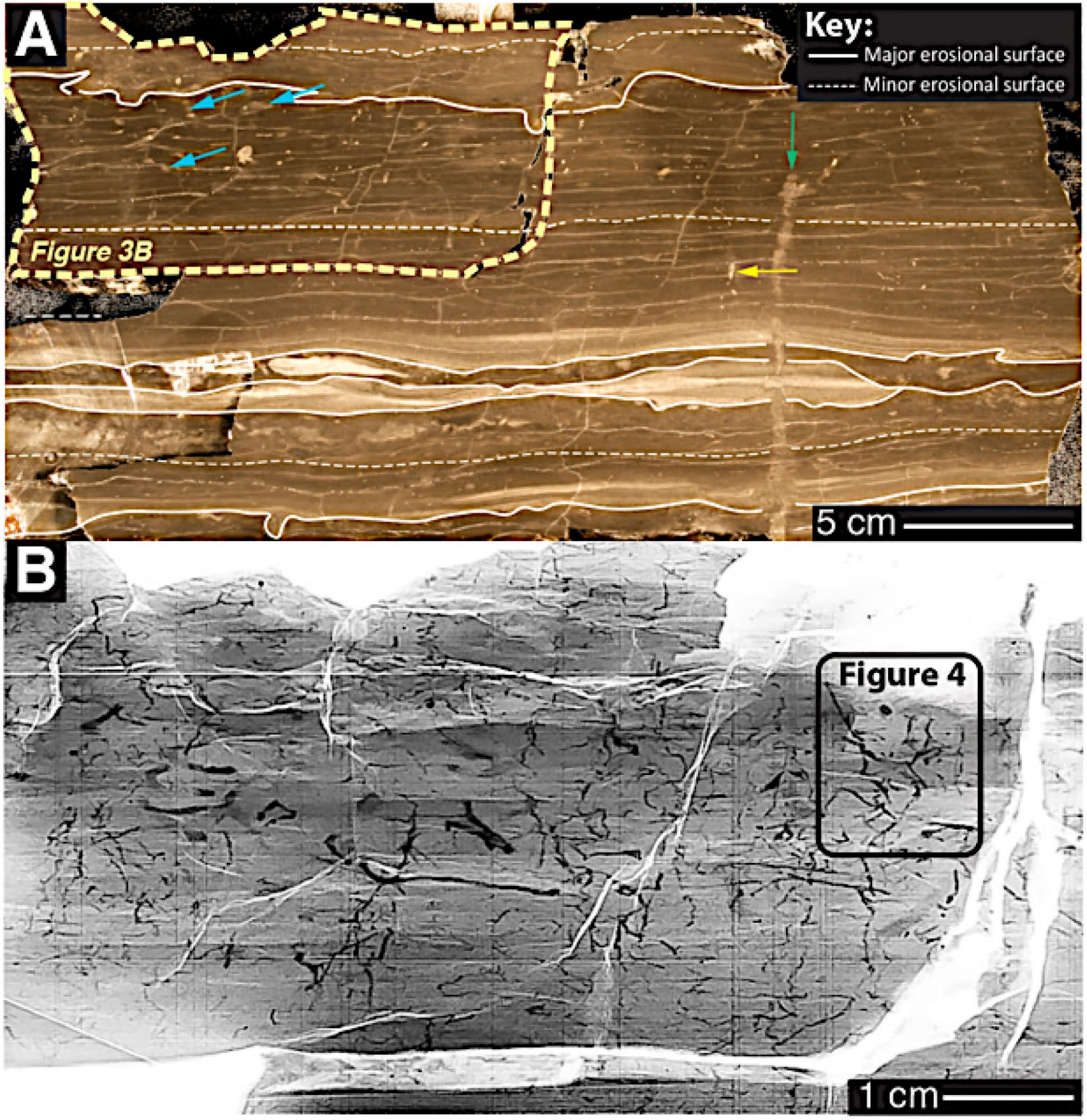
Sedimentologic context of pyrite formation in the Beach Formation. (**A**) shows moderately bioturbated mudstone composed of millimetre-thick beds with eroded tops. Much of the bioturbated mudstone exhibits deformed bedding and lamination due to animal locomotion (i.e., sediment swimming) in water-rich, non-compacted (possibly fluid?) mud. (**B**) shows the X-ray image of an enlarged subset of (A). This portion shows that the analyzed mudstone contains pervasive, vertically oriented, zigzagging, meandering pyritic trails and tubular structures (see Fig. 4 for more detail).

The findings of this study are based on an integration of the isotopic composition of sedimentary pyrite with its surrounding mudstone sediment texture and fabric (see Figs. 2, 3). The detailed sedimentology and ichnology of this mudstone was previously described in outcrop along with a set of large (20–30 cm in diametre) polished hand samples and thin sections^21^. The basal portion of the sampling locality Freshwater Cove (Fig. 2A) belongs to the shallow-marine Beach Formation, which is composed of heterolithic, laterally continuous, normally graded mudstone and sandstone beds^22^ (Fig. 2B). Approximately 13 m of the cliff exposure at Freshwater Cove have been logged at the centimetre scale (Fig. 2C). Ten whole-rock samples as well as two muddy siltstone beds, sampled at 21 m stratigraphic height (Fig. 2C) were selected for S- and Fe-isotope analysis. High-quality surface polish of large hand samples revealed a high number of erosion events between pyrite-rich intervals (Fig. 3A). Beds that contain pyrites utilized for analyses were interpreted as traction-dominated density flows with an initially high suspended silt concentration that was deposited under settling times of variable length^21^. In these beds abundant shallow-tier, palimpsest ichnofabrics rarely exceed a bioturbation index of 2 (BI = 0–2; 0–30%)^23^ (Fig. 3A, B). *Planolites* are preferentially concentrated at bed tops (Fig. 3A), while rare *Skolithos* cross-cut more than one event bed. Within more clay-rich portions, distorted biodeformational structures indicate foraging of worm-like animals in beds with initially higher water content compared to over- and underlying coarser-grained beds (Fig. 3B). The distorted biogenic structures within those clay-rich beds might represent exceptionally preserved examples of ancient fluid sediment swimmers^24^ (Fig. 3B).

## Materials and methods

A representative set of whole-rock samples (n = 10) were selected for δ^34^S and δ^56^Fe analyses via multi-collector inductively coupled plasma mass spectrometry (ICPMS) (Fig. 2). Measurements were performed on mudstones that contain a mixture of iron and sulfur-bearing phases besides pyrite. Fresh, non-weathered samples were separated from the rock face via hammer and chisel and were ground up for isotopic analyses. Approximately 1 g of sample material has been leached using 3 ml of *aqua regia*. The supernatant of that leaching process has been extracted after centrifugation. Two thirds of that volume has been used for S elution while the rest has been used for Fe elution (see supplementary file for analytical protocol).

High-resolution S- and Fe-isotope data were generated via secondary ion mass spectrometry (SIMS). All SIMS-based isotope measurements in this study were performed at the bed scale. This study does not mix pyrites from separate event beds and exclusively compares pyrite-δ^34^S and -δ^56^Fe along the same bed (Fig. 3). All samples were collected *in-situ* using hammer and chisel or a gas-driven rock saw to ensure recovery of fresh, non-weathered material. Microtomographic X-ray imaging of centimetre-sized rectangular samples of polished mudstone containing synsedimentary pyrite has been performed on a *phoenix nanotom s*, equipped with an X-ray nanofocus tube^25^. Sufficiently large (> 10 μm) pyrites suitable for SIMS analysis were cast in epoxy resin and diamond-polished resin mounts. The pyrites contained in the resin mounts were analyzed for ^32^S, ^33^S, ^34^S, ^54^Fe and ^56^Fe on a CAMECA IMS1280 secondary ion mass spectrometer (SIMS) at the NordSIM microprobe facility in Stockholm, Sweden (see supplementary information for detailed analytical protocol). The δ^34^S and δ^56^Fe measurements are reported in conventional notation relative to the international standards Canyon Diablo Troilite (CDT) for S (δ^34^S = 0.4 ± 0.05) and the average δ^56^Fe of igneous rocks, IRMM-14, with δ^56^Fe = −0.08 ± 0.08‰. Stable S and Fe isotope compositions are expressed as δ values using standard per mil (‰) notation, which is defined as:

$$\begin{aligned}\updelta ^{56} {\text{Fe}} &= \left( {\left( {\left( {^{56} {\text{Fe}}/^{54} {\text{Fe}}} \right)_{{{\text{sample}}}} /\left( {^{56} {\text{Fe}}/^{54} {\text{Fe}}} \right)_{{\text{IRMM - 14}}} } \right) \, - 1} \right) \cdot 1000 \hfill \\\updelta ^{34} {\text{S}} &= \left( {\left( {\left( {^{34} {\text{S}}/^{32} {\text{S}}} \right)_{{{\text{sample}}}} /\left( {^{34} {\text{S}}/^{32} {\text{S}}} \right)_{{{\text{VCDT}}}} } \right) \, - 1} \right) \cdot 1000 \hfill \\ \end{aligned}$$

Δ^33^S is a measure of deviation from a mass-dependent relationship, defined as:

$$\Delta^{33} {\text{S}} = 1000 \times \left[ {\left( {\frac{{1 + \delta^{33} S_{sample} }}{1000}} \right) - \left( {\frac{{1 + \delta^{34 } S_{sample} }}{1000}} \right)^{0.515} } \right]$$

## Results

### Geometries and morphologies of pyrites selected for SIMS analyses

**Figure 4:**
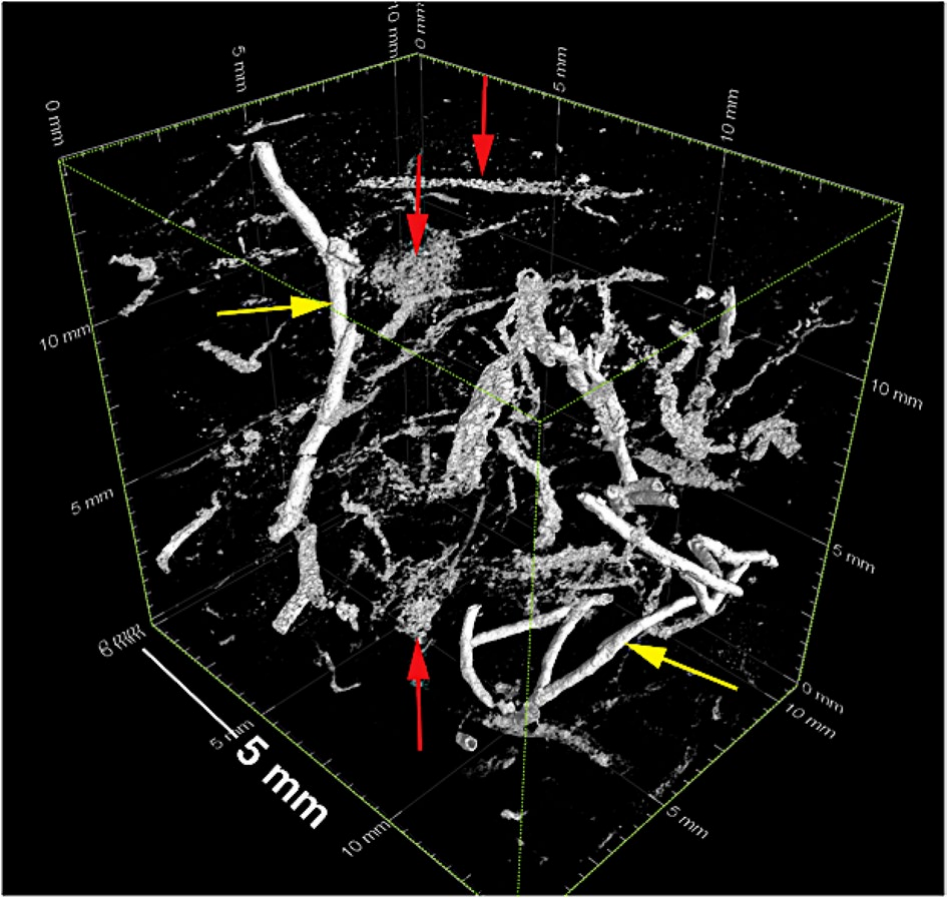
This subregion, marked with a black box in Fig. 3, has been selected for X-radiography. This higher-resolution scan reveals a three-dimensional framework of tubular pyritic structures (TPS—yellow arrows) as well as meandering, curved and zigzagging pyritic trails (PTs—red arrows) (see text for discussion).

**Figure 5:**
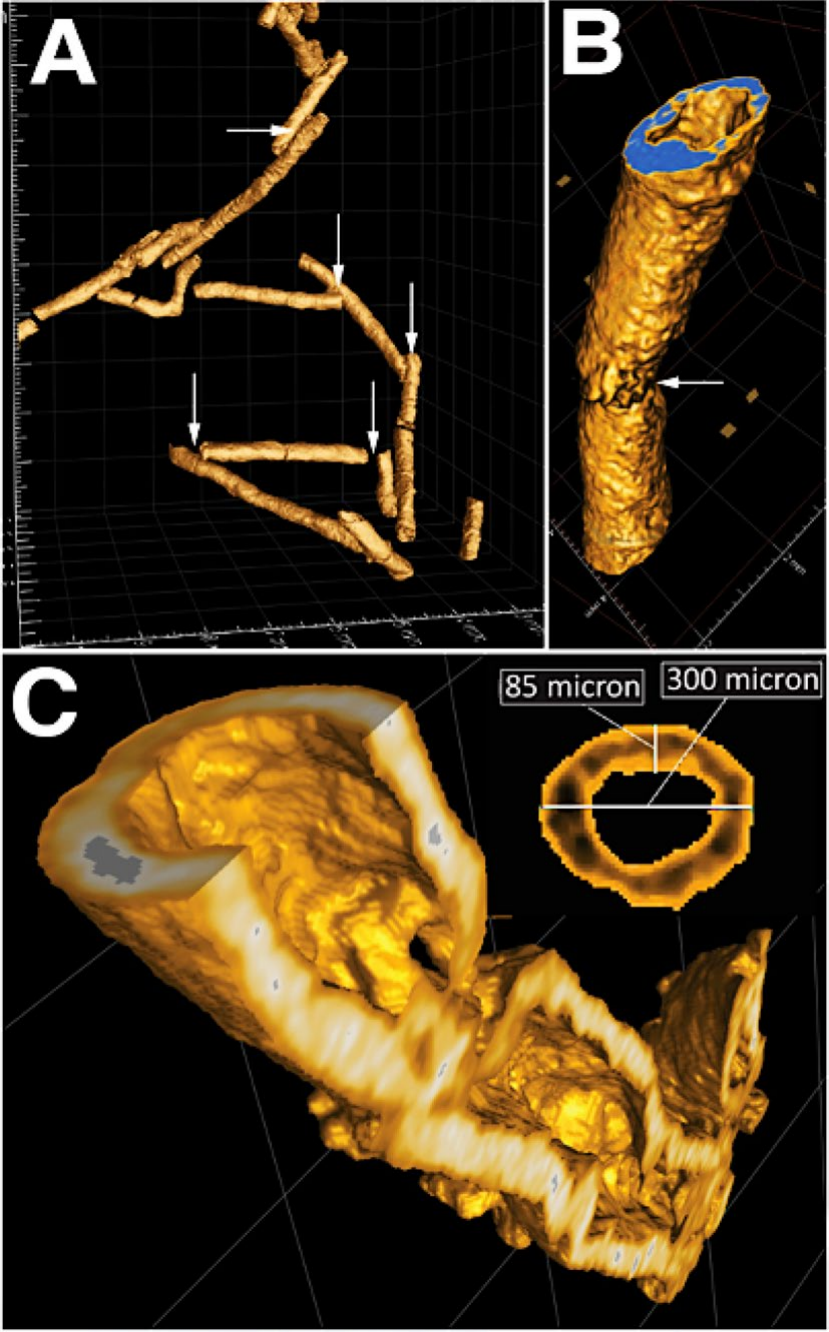
(**A**) Shows a filtered micro CT sub-volume highlighting the geometry and morphology of TPS (PTs were excluded this reconstruction). Note that TPS exhibit sheath-like morphology with collapse fabric that was most likely acquired during dewatering and bed compaction. The presence of well-preserved “knick points” (white arrows) indicates that TPS were constructed relatively early, prior to significant mud compaction. (**B**) close-up of a representative TPS. Note the well-developed knick separating two well-developed pyritic tubular bodies. (**C**) shows a vertical cross section through a representative TPS. Note the well-developed and continuous sheath morphology with a pyritic wall thickness of ~ 85 micron and a continuous overall diametre of ~ 300 micron.

Three types of sedimentary pyrite were selected for SIMS analyses: (1) Well preserved three-dimensional networks of tubular pyritic structures (TPS) and pyritic trails (PTs), (2) framboidal pyrite-filled *Planolites* burrows, and (3) single framboids from non-bioturbated mudstone. Extensive microtomographic X-ray imaging (Figs. 4, 5) reveals that TPS represent continuous and non-continuous, densely spaced, semi-vertical segmented or sheath-like pyritic tubular structures, which are ~ 200 to 300 μm in diametre (Fig. 5). TPS pyritic walls are exceptionally continuous, are ~ 80 to 100 μm thick and cross-cut several depositional events (Fig. 5). The PTs occur together with TPS but are discontinuous and appear curved to sinuous. PTs also show sharp turns and geometries resembling ‘zigzagging’ (Fig. 4). The host mudstone contains isolated pyrite framboids, which are neither associated with *Planolites* nor TPS and PTs. Those dispersed framboids were located via conventional petrography (see supplementary information for spot analysis setup).

### δ^34^S and δ^56^Fe isotope results

**Figure 6:**
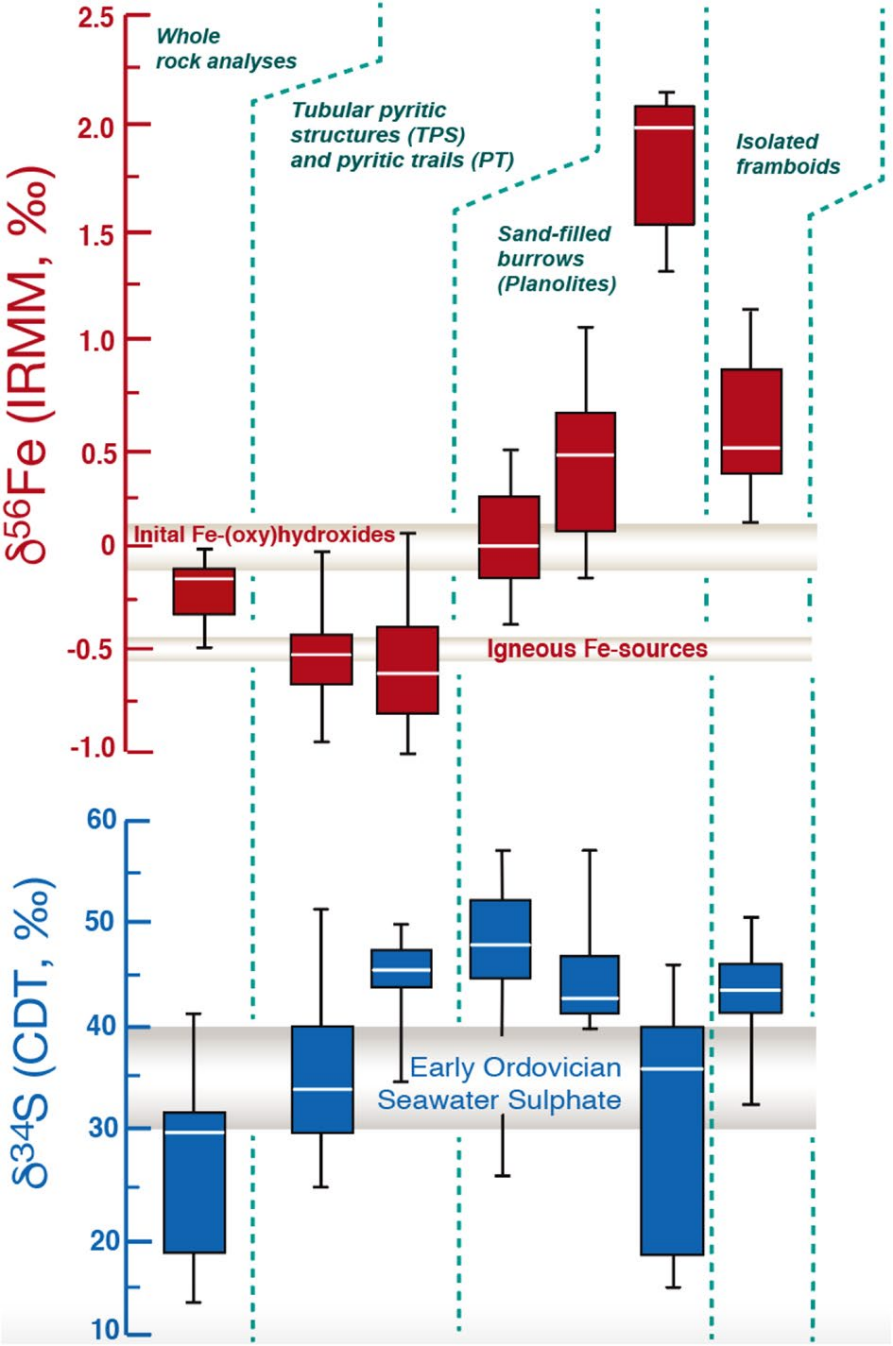
Box-and-whisker plots showing δ^56^Fe and δ^34^S SIMS results. Box-and-whisker plots were grouped based on sample and pyrite type and morphology – separating tubular pyritic structures (TPS) and pyritic trails (PTs) from sand-filled *Planolites* burrows and isolated framboids (see supplementary information for δ^33^S and △^33^S results).

**Figure 7:**
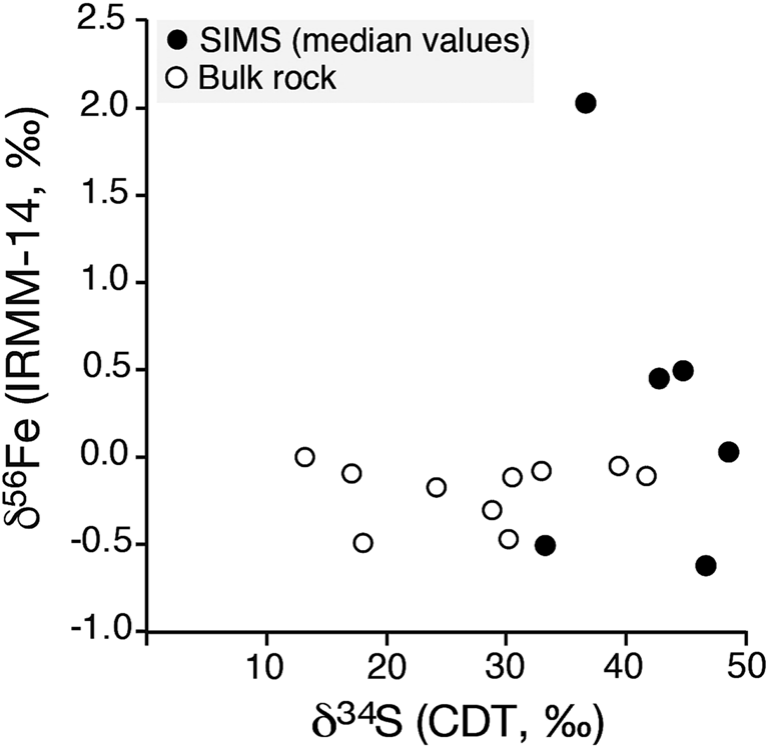
Cross-plot showing bulk-rock versus SIMS median (pyrite only) δ^56^Fe and δ^34^S values presented in this study. Note absence of a correlation between the two isotope systems for median SIMS-measured and bulk rock datasets.

In this study bulk rock δ^34^S values range between + 15‰ and 42‰, while the δ^56^Fe shows values between − 0.05‰ and − 0.5‰ (Table 1; Fig. 2). The isotope curve does not exhibit any trend throughout the succession and seems to reflect the stable isotopic composition of S and Fe parent phases. The SIMS-measured pyrite δ^34^S dataset covers a range between + 15‰ to + 65‰ (Table 1; Fig. 6) and is therefore more enriched in ^34^S than comparable sedimentary pyrites measured from bulk analyses in this study (Fig. 6) and elsewhere^2^. The SIMS-measured Δ^33^S range (− 0.1‰ to + 0.8‰) lies within the range of mass-dependent fractionation typical for an oxygenated atmosphere^26^. Both, TPS and PTs exhibit median values of δ^34^S ~ +40‰; δ^56^Fe ~ −0.5‰ and have been grouped within this study based on facies (Fig. 7). *Planolites* burrows reveal median values of δ^34^S ~ +40‰, too, but a wider range of median δ^56^Fe values ranging between ~ +0.5‰ to + 2.1‰ (Fig. 6). Dispersed pyrite framboids in the non-bioturbated sediment yielded δ^34^S ~ +35‰ and δ^56^Fe ~ +0.5‰ (Fig. 6).

**Table 1:** Statistics describing the isotopic composition of the studied iron sulfides.

Sample category	Sample	n	Minimum (‰)	First quartile (‰)	Median (‰)	Third quartile (‰)	Maximum (‰)	1σ (± ‰)
δ^34^S								
Pyritized sheaths and 'tubes'	FC1021	20	25.44	30.70	33.33	40.33	52.58	0.12
	FC1020-1	20	34.378	44.053	46.789	47.790	51.046	0.12
	FC1020-4	23	26.281	44.772	48.618	52.882	57.622	0.11
Sand-filled burrows	FC1020-6a	7	41.346	42.155	42.865	47.841	57.384	0.14
	FC1020-6b	5	16.171	19.266	36.731	40.549	46.479	0.11
Non-bioturbated	FC1020-7	6	33.141	42.191	44.817	47.063	50.902	0.16
δ^56^*Fe*								
Pyritized sheaths and 'tubes'	FC1021	19	− 0.92	− 0.68	− 0.52	− 0.43	− 0.08	0.10
	FC1020-1	17	− 1.00	− 0.80	− 0.63	− 0.38	0.16	0.10
	FC1020-4	25	− 0.34	− 0.13	0.02	0.25	0.94	0.09
Sand-filled burrows	FC1020-6a	9	− 0.13	0.10	0.44	0.67	1.08	0.20
	FC1020-6b	6	1.31	1.54	2.02	2.12	2.18	0.20
Non-bioturbated	FC1020-7	6	0.17	0.36	0.48	0.78	1.15	0.11
Beach Formation whole rock isotope analyses	Sample	Stratigraphic height (m)	δ^34^S‰ CDT (‰)	1σ (± ‰)	δ^56^Fe‰ IRMM-14 (‰)	1σ (± ‰)		
	FC1022	21.91	17.2	0.19	− 0.10	0.02		
	FC1020	20.96	41.7	0.15	− 0.12	0.08		
	FC1019	20.20	30.3	0.15	− 0.48	0.46		
	FC1018	19.45	28.9	0.46	− 0.31	0.21		
	FC1012	18.85	39.4	0.23	− 0.06	0.11		
	FC0915	18.15	18.1	0.20	− 0.50	0.18		
	FC1201	12.20	30.6	0.28	− 0.13	0.11		
	FC10XXX	11.73	24.3	0.22	− 0.19	0.01		
	FC09A5b	1.60	33.0	0.22	− 0.09	0.13		
	FC09A2b	0.85	13.3	0.41	− 0.01	0.15		

It shows the analytical δ^56^Fe and δ^34^S results for SIMS-measured pyrites as well as bulk rock obtained from the Beach Formation mudstones.

## Discussion

### Pyritic animal burrows—a gateway to ancient porewater chemistry?

In modern mud-rich sediment, animal burrows are key sites of sediment carbon remineralization due to the high volume of readily available reductants as well as inorganic oxidants^27^. Previous research has shown how pyritized animal burrows feature most prominently as reliable archives for the ancient low-temperature pore water cycling of sulphur and iron^11,12,28^. Animals who deploy burrowing as a feeding and life strategy, colonize the infaunal realm while the muddy sediment still contains sufficient amounts of dissolved oxygen^29–31^. Sediment ingestion, construction of dwelling structures and swimming through water-rich mud profoundly changes the chemical structure of the deposit itself. It breaks up the vertically structured, microbially-mediated early diagenetic reaction zonation^14,24^, and modifies the original permeability of the upper sediment layer^32^. Burrowing and sediment swimming itself does not only break-up this vertically structured reaction zonation but it also introduces ‘super-reactive’ organic carbon in the form of extracellular polysaccharides, or EPS^33^, into the sediment, which is locally highly abundant in the mucous lining of animal burrows^11^. Micro-organisms, such as sulfate reducing bacteria and archaea who are naturally occupying the burrow environment immediately oxidize this readily available EPS while reducing sulfate and iron to sulfides and dissolved iron species^14^. The now reduced sulfur and iron will combine to form FeS, which eventually converts to framboidal pyrite^34,35^. This framboidal pyrite preserves the unique isotopic composition of the pore water sulfide-δ^34^S and Fe(II)-δ^56^Fe, acquired prior to FeS precipitation^8^.

### Timing of pyrite precipitation

Detailed texture and fabric analyses of the Bell Island sandstone and mudstone demonstrate sediment deposition under strong (possibly seasonal?) storm reworking^22, 36^. Recent process sedimentological research carried out on the mud-dominated portion of the Beach Formation reveals abundant fabric evidence for wave and current-dominated deposition preserved in the form of wavy-discontinuous lamination, millimetre-thick graded beds and millimetre-thick sand- and siltstone ripples^21, 22^ (Fig. 3). These latter studies contest the traditional depositional model initially put forward^36^ that claims that the Beach Formation mudstones represent background sedimentation and post-storm settling of fines between storms.

Cross-cutting relationships between pyritic trace fossils versus sedimentation events (Fig. 3B) suggest that both, TPS and PTs, were constructed while oxygen was still present in the upper mud layer. The remarkable preservation (Figs. 4, 5) of the semi-vertical, sub millimetre-thick TPS are inferred to represent pyritized burrow linings of either meiofauna or small macrofauna of currently unknown origin^37,38^. Continuous TPS indicate originally open burrows (Fig. 5). The PTs, on the other hand, exhibiting less spatial continuity, are often randomly oriented and therefore interpreted to be the partially pyritized, EPS-rich burrow linings of meiofauna, which were foraging and swimming in soupy substrate^39^ (Fig. 4).

Non-pyritic, silt- and sandstone-filled, partially compacted *Planolites* burrows are only preserved as palimpsest ichnofabrics, preferentially close to bed tops (Fig. 3). We argue that those *Planolites* were formed after the formation of TPS and PTs, since they crosscut muddy event beds with fabric evidence for high-energy exhumation (eroded bed tops) and mud reworking (wavy-discontinuous silt laminae and sandstone-filled gutters)^21^ (Fig. 3A). Interestingly, even after TPS, PTs and non-pyritic *Planolites* have been constructed, the sediment is inferred to still have been plastic enough to allow for the construction of vertical, decimetre-long sandstone-filled *Skolithos* burrows (Fig. 3A).

### Reactants for pyrite formation

Localized sedimentary pyrite formation requires the presence and supply of a reductant, such as EPS-hosted organic matter (in some cases methane can also function as reductant) to reduce sulfate to sulfide, and bioavailable ferric iron (i.e., α-FeOOH and Fe_2_O_3_) to dissolved Fe(II)^40^. The pervasive presence of pyritic meiofaunal burrows (TPS and PTs) throughout analyzed mudstone event beds (Figs. 3, 4) indicate an initially high availability of reactive sulfur and iron in pore waters, and locally elevated reactive organic carbon. The spatial distribution of bioavailable organic matter cannot have been equal throughout the entire Beach Formation, given the near absence of pyrite away from the bioturbated zone. Effectively, reactive organic matter must have been confined to small animal burrows and to spatially constrained wavy stringers of organic matter, such as microbial mats^20^. Measured δ^13^C_org_ data for this part of the Beach Formation reveal that the majority of the deposited organic carbon is of marine origin (δ^13^C_org_ range − 22 to − 26‰)^20^. Petrographic examination of the Beach Formation kerogen demonstrated that only layers with microbially-sourced organic matter and TOC values above 1.0 wt.% contain locally elevated amounts of framboidal pyrite^20^. The remainder of the Beach Formation mudstones is fairly lean in organic matter (< 1.0 wt.% TOC)^21^.

Microbially-driven fractionation of reduced sulfur and carbon species are invoked to explain the starkly enriched pyrite δ^34^S trends above ambient seawater sulfate, as well as the high disparity in pyrite δ^56^Fe between two different pyrite groups. Continuous storm reworking of the Beach Formation coastal muds and sands must oxidize any high-quality marine organic carbon and render it less reactive^41^. Based on the presence of high volumes of pyrite in animal burrows, it is proposed that TPS must have initially contained high concentrations of EPS, which were rapidly utilized by microorganisms. Sediment-colonizing animals must have, at least in the beginning, taken advantage of the fact that oxygen was abundant throughout the pore waters of the entire muddy event bed. Mucous-lined meiofaunal burrows could have functioned, initially, as the only sulfate-reduction ‘hot spots’ within an organic carbon-lean sediment matrix^10,11,42^. The difference in extent of pyrite mineralization between PTs and TPS, however, indicates the presence of differing volumes of burrow-hosted EPS. Microbial colonies probably oxidized organic carbon and reduced reactive Fe oxides adjacent to the mucous lining of the meiofaunal burrow, leading to the observed patchy pyritization (Fig. 4), whereas the prominent, impervious and continuous pyritic sheaths of TPS (Fig. 4) suggest either longer residence times in a given sediment portion (with concomitant EPS secretion) or the availability of additional reductants such as methane or hydrogen sulfide. The supply of methane, in particular, would have continued to fuel the reduction of sulfate and Fe-oxides after the burrowing animals abandoned their still open, but by that time, anoxic burrow microenvironment^18,43^.

The Beach Formation mudstones were originally sourced as a highly immature assemblage of only partially-weathered volcanic lithoclasts from an Early Paleozoic, non-vegetated hinterland^21^. In conjunction with the low measured TOC values (the majority of samples is well below 1.0 wt.%)^21^, suggests that early Paleozoic mudstones at this specific location originally contained little bioavailable organic matter, except in sediment portions in which mucous-lined burrows provided high-quality EPS for microbial sulfate and iron reduction. A possible alteration of pyrite-hosted stable S and Fe isotope signatures via hydrothermal, sulfide-rich fluids can be excluded, since such an alteration would have resulted in the precipitation other sulfur phases (i.e., barite, metal sulfides) throughout the sediment matrix^19,44^.

### Evidence for ‘closed system’ diagenesis in the Beach Formation?

On geologic time scales pyrite is the most stable iron disulfide and represents the low-temperature end product of sulfur and iron cycling in marine sediment^40^. The isotopically enriched SIMS-measured pyrite-δ^34^S (on average ~ +40‰) measured within this study ranges well within the inferred sulfate δ^34^S values of contemporary Early Ordovician seawater sulfate (between + 30‰ and + 40‰)^45^. Since no known microbial pathway is capable of enriching ^34^S in iron disulphide above its parent phase in ambient seawater, the abnormally high isotopic enrichment of pyrite-δ^34^S within this study can have only proceeded via a multi-step fractionation tied to closed system diagenesis^46^. The poor overlap between SIMS-analyzed and whole rock δ^56^Fe and δ^34^S (Fig. 6) supports the presence of micro reservoir effects driving the isotopic variability of pyrite at the bed scale.

Significant enrichment in ^34^S has recently been measured in pyrite that formed in water-rich muds deposited of modern tropical shelves under slow sediment accumulation rate^47^ and pronounced methane seepage^17,18,48,49^. In these modern-day slow sedimentation settings, the availability and supply of bioreactive carbon in the form of methane is unlimited. If sulfate is not quickly enough replenished in pore waters, then the pore-water concentration of sulfate is constantly shrinking. Given that microbial cells preferentially incorporate isotopologues with ^32^S, means that the relative concentration of ^34^S will increase within the porewater sulfate reservoir^50^. If this fractionation process continues, then the δ^34^S composition of pyrite precipitating from this sulfate reservoir will eventually begin to approach the seawater sulfate-δ^34^S and go even beyond the parent phase value^46^ (see Fig. 7).

Well-pronounced TPS “collapse” geometries (Fig. 5A) indicate that the host sediment must have experienced significant compaction and porosity reduction (Fig. 5A). This highlights that TPS were constructed prior to compaction, and potentially functioned as conduits for upward seeping methane^17^. However, no independent evidence for methane seepage (e.g., vertical calcareous chimneys, stratabound concretionary intervals) has been observed in the vicinity of the sampling site. Instead, with limited proposed sulfate pore water concentrations^51^, large microscale reservoir effects (i.e. Rayleigh-type distillation)^50^ are more likely to have depleted ^32^S in these burrow microenvironments. It is currently unknown how permeable the freshly deposited host sediment was, but given its muddy nature, probably not very. Micro-reservoir effects, prevalent in such an environment could have been sufficiently strong for some burrow microenvironments allowing the generated H_2_S-δ^34^S to successively approach the enriched seawater S-δ^34^S, regardless of the magnitude of fractionation^46^.

### A relationship between physical seafloor remobilization and S isotopic enrichment in pyrite?

A mixed layer encompasses the uppermost, bioturbated centimetres of marine sediment^52^. The upper part of the mixed layer is usually oxygenated and permeable. With the presence of initially high volumes of reactive organic matter, it accounts for the generation of high volumes of microbially derived metabolites^30,53^. Low bioturbation indices (BI 0–2; 0–33%)^54^ and exceptional preservation of primary sedimentary structures down to the millimetre-scale throughout the early Ordovician Beach Formation (Harazim et al., 2013, 2015) demonstrate that this mudstone, initially, did not possess a fully-developed mixed layer. Similar to many other preserved early Palaeozoic depositional systems in Newfoundland^52,55,56^, the uppermost millimetres of the sediment in the Beach Formation muds were most likely firm. Consequently, the removal of upper sediment layers during storms would have generated physically disturbed dysoxic top layers, that were separated by sharp redox discontinuities against anoxic bottom layers^57^. 1-D reaction transport modeling in modern physically disturbed depositional systems shows that repeated sediment remobilization promotes isotopic S fractionation, such as in the Amazon-Guianas coastal muds^23^. If reactive, dissolved Fe is available, then diffusion of sulfate from the upper, dysoxic sediment layer into the underlying, anoxic one would lead to its conversion to hydrogen sulfide and, subsequently, to pyrite precipitation in the anoxic sediment. A key tenet of this modern, physically disturbed environment is that the precipitated pyrite on average becomes progressively more enriched in ^34^S, the more often the seafloor reworking is repeated^23^. A similar scenario could be envisioned for the Beach Formation. If any partial oxidation of H_2_S and its subsequent reduction^58^ were to be repeated several times during storm reworking, then, in total, the concentration of ^34^S within porewaters would be elevated, and eventually, precipitate pyrite with δ^34^S values higher than that of the Early Ordovician contemporaneous seawater sulfate, as observed in this dataset (Fig. 6). More isotope studies, tightly knit into a high-resolution stratigraphic framework will provide insight as to which process better explains the differences between pyrite-bound S and Fe.

### Proposed origin of pyrite-δ^56^Fe enrichment in *Planolites* burrows

The SIMS-measured TPS and PTs pyrite-δ^56^Fe values show insignificant fractionation from its bulk rock counterpart (Fig. 6) and a globally defined magmatic Fe source (δ^56^Fe ~ −0.5‰)^59^. In contrast, δ^56^Fe values in *Planolites* are much higher (+ 2.1‰) (Fig. 6). Pyrite-δ^56^Fe can exhibit significant enrichment above parent phase signatures when formed within small animal burrows, because in those burrows the presence of organic carbon-rich EPS fuels very high rates of Fe fluxes associated with microbial iron reduction that quickly exhaust the local ^54^Fe pool after FeS begins to precipitate^12^.

**Figure 8:**
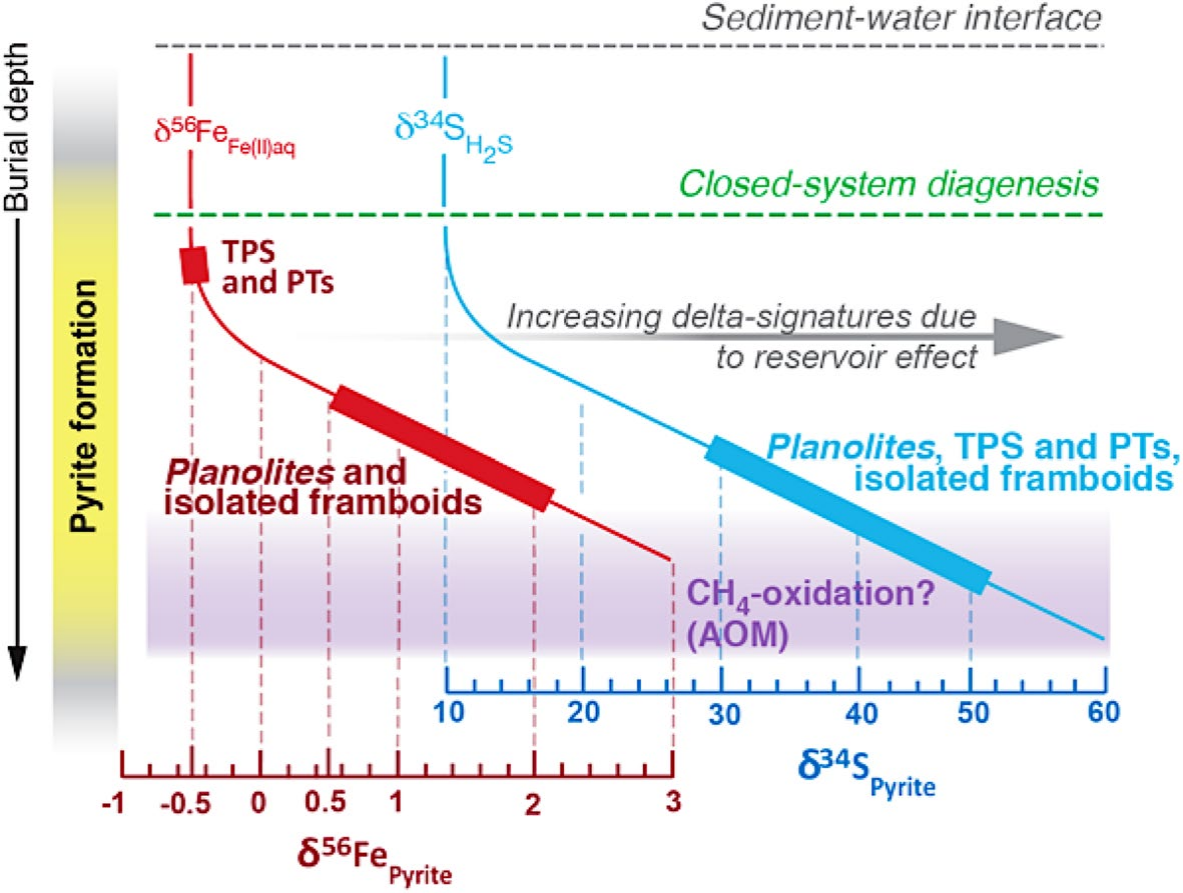
This block diagram proposes a pathway for generating disparate δ^56^Fe and δ^34^S values between TPS, PTs and isolated framboids. We consider a scenario in which pyrites form from a finite reservoir of pore water hydrogen sulfide as well as dissolved ferrous iron. Early precipitating pyrite will acquire isotopic signatures that represent a natural kinetic fractionation effect. In the case of δ^34^S, bacterial sulfate reduction generates H_2_S-δ^34^S that is approximately 30‰ lighter than seawater sulfate-δ^34^S (Early Ordovician seawater ~ +40‰^45^). In accordance with this mechanism, the dissolved Fe-δ^56^Fe will be on average − 0.5‰ lighter compared to the seawater dissolved Fe-δ^56^Fe, which is proposed to lie around 0.0‰. The heavy isotope ranges for both iron and sulfur are typical for Raleigh-type fractionation within a closed diagenetic system (see text for discussion).

In the early Ordovician Beach Formation it is inferred that *Planolites* trace makers most likely resemble small worm-like organisms that excavated and ventilated their burrows within a partially consolidated, firm substrate^52,60^ whereas the much smaller TPS and PT trace makers, at an earlier point in time, exploited the still soupy, fully oxygenated muddy substrate. Within silty *Planolites* burrows, the fractionation in dissolved Fe(II)-δ^56^Fe has been more severe than in pyrite-δ^34^S. It is argued that iron diffused into *Planolites* burrows possibly at a much slower rate compared to sulfide, thereby being highly susceptible to severe microscale reservoir effects and yielding pyrites with starkly enriched pyrite-δ^56^Fe signatures, well above + 0.5‰^12, 61^ (Fig. 6). Alternatively, a shallow sulfate-methane transition zone^16^ could have promoted the enhanced stripping of ^54^Fe from pore waters and preferred incorporation into the first precipitated pyrite. Physical reworking and associated abiotic oxidation of the remaining dissolved Fe(II) pool immediately after bed reworking could have also shifted Fe(II)-δ^56^Fe further towards heavier end-members and lead to local enrichment of above + 2.0‰ during pyrite precipitation (Fig. 8). Further studies will corroborate the proposed relationship between microbial iron reduction, microscale reservoir effects and the isotopic signatures of sedimentary pyrite in animal burrows.

### Implications for understanding earth’s past iron and sulfur cycle

In bioturbated mudstones, microscale reservoir effects can significantly enrich the stable Fe and S isotope signatures of sedimentary pyrite and impart significant stratigraphic variability in bulk S and Fe isotope datasets—without the need to invoke perturbations in the global ocean-scale reservoir (Fig. 8). Any geochemical measurement from sedimentary rocks exhibit significant noise, due to difference in composition between single beds and even laterally across one single bed^62^. A key future research direction will constitute of better linking the diagenetic history of a sedimentary unit with its stable isotope signature. Most importantly, commonly deployed whole-rock sampling techniques that homogenize discrete depositional events should be avoided. At present, more and more studies^46,63^ realize that if stable S and Fe isotopes are to be employed as high-fidelity recorders of past paleoenvironmental conditions, then understanding the origin and high degree of uncertainty must be a key topic and at the forefront of stable isotope research. At its heart, it must be conducted as a microscale approach that honors all original stratigraphic relationships between different depositional and diagenetic events and does not mix pyrites of dissimilar origin^12,64^. The magnitude of ^34^S and ^56^Fe enrichment in pyrite is dependent on a variety of sedimentological factors and diagenetic drivers, such as sedimentation rate and reworking frequency, amount of pyrite re-oxidation, S- and Fe-reduction rate, methane supply to the sulfate-methane transition zone, as well as availability of interstitial reactive iron^7, 49^ (Fig. 6). The continuous discovery of new microbially mediated energy harvesting metabolisms associated with methane oxidation, especially the ones that involve the reduction of more crystalline iron-rich clay minerals and iron oxides via anaerobic oxidation of methane (‘Fe-AOM)^65^ as well as long-distance electron transfer via the recently discovered cable bacteria^66^ promise to further our understanding and importance of isotope fractionation for diagenetic processes not in accordance with the traditional Froelich sequence^67^. In our opinion, the greatest discoveries will emerge from integrated sedimentological-stable isotope studies which are going to reveal how widespread and common the non-steady redox cycling of sulfur and iron is in deep time^19^ (Fig. 1). Ancient, high-energy muddy coastlines are notoriously difficult to recognize from geological datasets. Only recently sedimentologists have begun to erect recognition criteria for bedload transport of mud and wave- and current-dominated seafloor reworking^21,68–73^, while the competing diagenetic pathways in those successions are not yet fully understood at the scale at which they are most pronounced (the millimetre to centimetre-scale). More texture-specific, combined S and Fe isotopic studies will corroborate the here proposed relationship between (1) microscale reservoir effects, (2) methane and/or sulfide oxidation and (3) high-frequency redox cycling associated with bioturbation and surface sediment reworking. Only then the community will begin to quantify how much of the noisy, secular stable S and Fe isotope proxy record analyzed to date (see Fig. 1) can be assigned to localized, early diagenetic cycling of S and Fe, and how much of this variability is truly driven by processes operating at the scale of the global ocean–atmosphere reservoir^74^.

## Conclusions

This contribution presents a unique pyrite-δ^34^S and δ^56^Fe record from an excellently preserved early Ordovician, shallow-marine, muddy coastline that yields insight into the highly dynamic workings of the shallow-marine, anoxic iron and sulfur cycle. Pyrite-hosted δ^34^S and δ^56^Fe were analyzed from bioturbated and non-bioturbated storm-dominated, muddy coastline deposits of the early Ordovician Beach Formation (Newfoundland). Texture-specific SIMS analyses of exceptionally preserved tubular pyritic structures, pyritized trails and pyrites from sand-filled *Planolites* burrows reveal highly enriched δ^34^S (30–50‰) and an enrichment in δ^56^Fe between + 0.5 to + 2.1‰. The measured stable S isotopic signatures are within and slightly above the previously published early Ordovician ambient seawater δ^34^S values, while pyrite-δ^56^Fe show significant enrichment (~ +2.1‰) compared to published iron parent phases in the sediments.

Based on strong sedimentological evidence for high-energy deposition during pyrite formation and the sequential fractionation of S and Fe during pyrite precipitation within animal burrows, it is proposed that stable sulfur and iron isotope enrichment above parent phase values likely highlights the presence of micro scale reservoir effects and incorporation of the heavier ^34^S and ^56^Fe from the residual pore waters. Stark enrichment of δ^56^Fe values in pyrite in *Planolites* burrows by more than + 2.0‰ compared to earlier-formed pyritic burrows (TPS and PTs; δ^56^Fe ~ −0.5‰) highlights the progressive depletion of ^54^Fe in the pore water and potentially in the parent phases. The δ^34^S values measured from the same pyrite do not show comparable differences because the sulfate required for the sulfide production was more readily available in the porewater than Fe(II), which was released by the microbial reduction of iron-rich solid parent phases.

This study provides a gateway into the study of other, highly noisy deep time stable Fe and S isotope proxy records. High-energy seafloor processes in ancient mudstone-dominated deposits might prove as the preferred environment of formation for so-called ‘superheavy pyrite’^49^, a pyrite variety with stable isotope values above parent phase values that potentially accounts for the large shifts and variability measured in stable S and Fe isotopes throughout the Phanerozoic and Proterozoic rock record.

## Supplementary information

Supplementary information.
