# Supplementary material for: Exceptional sulfur and iron isotope enrichment in millimetre-sized, early Palaeozoic animal burrows
Source: Sci Rep. 2020 Nov 20;10:20270. doi: 10.1038/s41598-020-76296-8 (PMC7679392; doi:10.1038/s41598-020-76296-8)
Supplement: Supplementary file 1 — Supplementary information. [file 41598_2020_76296_MOESM1_ESM.pdf]

Supplementary Files to:

# Exceptional sulfur and iron isotope enrichment in millimetre-sized, early Palaeozoic animal burrows

Dario Harazim[1,2*], Joonas J. Virtasalo[3], Kathryn C. Denommee[4], Nicolas Thiemeyer[5], Yann Lahaye[6], Martin J. Whitehouse[7]

[1]Department of Geosciences, University of Calgary, 2500 University Drive NW, Calgary, AB, Canada, T2N 1N4.

[2]Department of Geology and Geophysics, Louisiana State University, E235 Howe-Russel-Kniffen Geoscience Complex, Baton Rouge, LA 70803, U.S.A.

[3]Geological Survey of Finland (GTK), P.O. Box 96, FI-02151 Espoo, Finland

[4]ExxonMobil Upstream Research Company, 22777 Springwoods Village Parkway, Spring, TX, 77389, U.S.A.

[5]Viscom AG, Carl-Buderus-Strasse, 6-15, 30455, Hannover, Germany

[6]Finland Isotope Geoscience Laboratory, Geological Survey of Finland (GTK), P.O. Box 96, FI-02151 Espoo, Finland

[7]Department of Geosciences, Swedish Museum of Natural History, Box 50007, SE-104 05 Stockholm, Sweden

This document contains:

1. SIMS microscale S and Fe isotope analyses and ICPMS whole-rock S and Fe isotope analyses

2. The tabulated SIMS-measured $\delta^{56}$Fe and $\delta^{34}$S results of the studied iron sulfides.

3. Whole-rock measured $\delta^{56}$Fe and $\delta^{34}$S results from Freshwater Cove, Bell Island, NL.

4. High-resolution photomontages of selected iron sulfides from sample assemblage FC1020-6 (Puck D).

**Secondary ion mass spectrometry – microscale S and Fe isotope analyses**

Microscale isotope compositions of iron sulfide minerals in gold-coated samples were analysed at the NordSIM facility in Stockholm, Sweden, using a Cameca IMS1280 secondary ion mass spectrometer (SIMS) operated in multicollector mode. Analytical methods and instrument parameters for the S-isotope determinations were broadly similar to those described by Whitehouse et al. (2005); briefly, a +10 kV, c. 1.5 nA 133$Cs^+$ primary beam, producing a c. 6 μm spot, was operated together with a normal incidence electron flooding gun for charge compensation, and a –10 kV secondary beam. Magnetic field stability was maintained using NMR regulation. Secondary ion signals of $^{32}$S, $^{33}$S and $^{34}$S were detected simultaneously in three Faraday detectors. The 33S signal provided an additional quality control on the data via observed colinearity on the mass dependent fractionation line in a three-isotope plot ($^{33}$S/$^{32}$S vs. $^{34}$S/$^{32}$S), which rules out any possibility of a detector bias on one or more of the individual signals. Pyrite isotope standards (Ruttan and Balmat pyrites; Crowe and Vaughan, 1996) were mounted together with each sample and analysed at regular intervals during automated sample sequences. In other S-isotope investigations in the Nordsim laboratory using these standards (e.g. Whitehouse, 2013),

some isotopic heterogeneity has been observed in the Balmat standard, and for this reason all data have been normalized only to the Ruttan standard with $\delta^{34}SCDT$ = 1.408‰ (Cabral et al., 2013). External reproducibility on $\delta^{33}S$ and $\delta^{34}S$ for standards in each analytical session after propagating the within run and external uncertainties was typically ±0.2‰ (1 standard deviation). The methodology used for Fe-isotope measurement closely flows that described by Whitehouse and Fedo (2007) but with minor changes to the primary beam size and transmission parameters (Virtasalo et al., 2015) that are additionally detailed here. A –13kV $O_2^-$ primary beam was projected through a 100 µm primary aperture to yield a c. 10 µm analytical spot on the sample with c. 2 nA beam current. Secondary ions were extracted at +10 kV using high transmission mode optics (160× field magnification, with an image field of c. 30 µm in a 5000 µm field aperture). The species $^{54}Fe+$ and $^{56}Fe+$ were measured simultaneously in two Faraday detectors on the IMS1280 multicollector array. In order to monitor any potential isobaric interference on $^{54}Fe$ from $^{54}Cr$, $^{52}Cr+$ was also measured in an ion counting electron multiplier; no significant counts were detected from the pyrites in this study. All detectors were operated at a mass resolution (M/ΔM) of 2560. A NMR field sensor locked the magnetic field with high precision, permitting a wider entrance slit (120 µm) than used by Whitehouse and Fedo (2007); as a result, secondary ion count rates and internal precisions were comparable despite the smaller primary beam utilized in this study. Balmat pyrite ($\delta^{56}Fe$ = –0.40 ±0.04‰, 2σ; Whitehouse and Fedo, 2007) was used as the primary standard, and Isua pyrite 248474 ($\delta^{56}Fe$ = +0.94 ±0.25‰, 2σ; Whitehouse and Fedo, 2007) as a monitor. External reproducibility on $\delta^{56}Fe$ in each session was typically ±0.1‰ (1 standard deviation). Isotope data were acquired over eight sessions (four for S, four for Fe), each of which comprised the unknown targets interspersed regularly with analyses of the primary reference. All analyses were performed in fully automated chain sequences with pre-sputtering for 90 seconds to

remove the Au coating over an area of c. 25×25 μm followed by centering of the secondary beam in the field aperture to account for the 67 effects of small surface relief. Each analysis consisted of 16 cycles of 4 second integrations. Values below the sample/standard count rate ratio of 0.6 were rejected from further analysis. This threshold is on the safe side because isotope ratios previously determined by SIMS in the Nordsim facility have been noted to suffer bias below the ratio of 0.5 (Virtasalo et al., 2013). The results for each analyzed spot are listed in Tables 1, 2 and 3.

**Multi-collector ICPMS – whole-rock F and S isotope analyses**

Secondary ion mass spectrometry – whole-rock F and S isotope analyses 1g of sample has been leached using 3ml of *aqua regia*. The supernatant of that leaching process has been extracted after centrifugation. Two thirds of that volume have been used for S elution while the rest has been used for Fe elution:

*Sulfur:*

The samples have been eluted following the classic liquid column chromatography technique described in Paris (2013), omitting the addition of NaCl. The samples have been diluted into a disposable 2ml beaker in 1.0 to 1.5 ml HNO3 2% down to the concentration of 20 ppm. The analyses were carried out by using a 50μl PFA MicroFlowTM nebulizer and a Multi-Collector Inductively Coupled Plasma Mass Spectrometer (Nu InstrumentsTM) at high mass resolution ($\Delta m/m = 3000$). The S isotopic measurements were performed in static mode using three faraday detectors, and 1 block of 60 integrations of 8 s. A blank has been measured in between every samples. The total procedural blank (chemistry plus instrumental) was about 19ppb S. The results

on the IAEA standard S-3 is - 32.2 ± 0.4 (2s, n=8) ‰, while the recommended value is -32.488 ‰.

*Iron:*

The samples have been eluted following the column chromatography method described in Craddock & Dauphas (2011). Iron was separated from matrix elements by anion exchange chromatography (Bio-Rad; AG1-X8 200-400 mesh) in a HCl medium. The digested sample was loaded in 0.5ml conc HCl in which iron was quantitatively retained on the resin and matrix elements were eluted using 5ml HCl conc, then 22ml 4M HCl, then 1ml 0.4M HCl. Iron was subsequently eluted from the resin using 15ml 0.4 mol l-1 HCl. That elution was repeated twice. An on-peak zero correction was use before every analysis and the isotopic reference material (IRMM-014) was used for sample bracketing. The international basaltic standards BCR2 and BHVO have been used for quality control.

**References**

Cabral, R. A., Jackson, M. G., Rose-Koga, E. F., Koga, K. T., Whitehouse, M. J., Antonelli, M. A., ...& Hauri, E. H. (2013). Anomalous sulphur isotopes in plume lavas reveal deep mantle storage of Archaean crust. Nature, 496(7446), 490-493.

Craddock, P.R. and Dauphas, N., 2011, Iron Isotopic Compositions of Geological Reference Materials and Chondrites: Geostandards and Geoanalytical Research, v. 35, p. 101-123.

Crowe, D.E., and Vaughan, R.G., 1996, Characterization and use of isotopically homogeneous standards for in situ laser microprobe analysis of 34S/32S ratios: American Mineralogist, v. 81, p. 187–193.

Paris, G., A.L. Sessions, A.V. Subhas, and J.F. Adkins, 2013, MC-ICP-MS measurement of δ34S and Δ33S in small amounts of dissolved sulfate: Chemical Geology, v. 345, p. 50-61.

Virtasalo, J.J., Whitehouse, M.J., and Kotilainen, A.T., 2013, Iron isotope 114 heterogeneity in pyrite fillings of Holocene worm burrows: Geology, v. 41, p. 39–42.

Virtasalo, J.J., Laitala, J.J., Lahtinen, R., and Whitehouse, M.J., 2015, Pyritic event beds and sulfidized Fe (oxyhydr)oxide aggregates in metalliferous black mudstones of the Paleoproterozoic Talvivaara formation, Finland. Earth and Planetary Science Letters, v. 432, p. 449–460.

Whitehouse, M.J., and Fedo, C.M., 2007, Microscale heterogeneity of Fe isotopes in >3.71 Ga banded iron formation from the Isua Greenstone Belt, southwest Greenland: Geology, v. 35, p. 719–722.

Whitehouse, M.J., Kamber, B.S., Fedo, C.M., and Lepland, A., 2005, Integrated Pb- and S isotope investigation of sulphide minerals from the early Archaean of southwest Greenland: Chemical Geology, v. 222, p. 112–131.

Whitehouse, M.J., 2013, Multiple sulfur isotope determination by SIMS: Evaluation of reference sulfides for Δ33S with observations and a case study on the determination of Δ36S: Geostandards and Geoanalytical Research, v. 37, p. 19–33.

**Table 1. Results of the ion microprobe sulfur isotope analyses**

| Sample name | Sample ID | Seq. in run | 32S cps (x 109) | 32Clsamp/av.std | 34S/32S drift corrected | ± abs | δ34S samples | ± ‰ |
|---|---|---|---|---|---|---|---|---|
| Session 1 - Puck A | | | | | | | | |
| FC1021-2 | PuckA_mt1189_A1@1 | 7 | 0.900 | 1.1243 | 0.045603 | 0.000005 | 29.44 | 0.13 |
| FC1021-2 | PuckA_mt1189_A1@2 | 8 | 0.891 | 1.1131 | 0.045831 | 0.000006 | 34.60 | 0.15 |
| FC1021-2 | PuckA_mt1189_A1@3 | 9 | 0.902 | 1.1271 | 0.045591 | 0.000002 | 29.17 | 0.09 |
| FC1021-2 | PuckA_mt1189_A1@4 | 10 | 0.890 | 1.1115 | 0.046170 | 0.000003 | 42.24 | 0.10 |
| FC1021-2 | PuckA_mt1189_A1@5 | 11 | 0.859 | 1.0725 | 0.046306 | 0.000003 | 45.32 | 0.11 |
| FC1021-2 | PuckA_mt1189_A1@6 | 12 | 0.920 | 1.1497 | 0.045757 | 0.000002 | 32.92 | 0.09 |
| FC1021-1 | PuckA_mt1189_A2@7 | 15 | 0.827 | 1.0331 | 0.046628 | 0.000002 | 52.58 | 0.09 |
| FC1021-1 | PuckA_mt1189_A2@8 | 16 | 0.862 | 1.0769 | 0.045646 | 0.000004 | 30.41 | 0.11 |
| FC1021-1 | PuckA_mt1189_A2@9 | 17 | 0.854 | 1.0670 | 0.046347 | 0.000008 | 46.24 | 0.20 |
| FC1021-1 | PuckA_mt1189_A2@10 | 18 | 0.854 | 1.0668 | 0.046155 | 0.000004 | 41.92 | 0.12 |
| FC1021-1 | PuckA_mt1189_A2@11 | 19 | 0.840 | 1.0498 | 0.046460 | 0.000002 | 48.80 | 0.09 |
| FC1021-1 | PuckA_mt1189_A2@12 | 20 | 0.838 | 1.0471 | 0.046087 | 0.000002 | 40.37 | 0.08 |
| FC1021-3 | PuckA_mt1189_A3@13 | 25 | 0.777 | 0.9711 | 0.045797 | 0.000005 | 33.83 | 0.13 |
| FC1021-3 | PuckA_mt1189_A3@14 | 26 | 0.823 | 1.0275 | 0.045747 | 0.000002 | 32.69 | 0.08 |
| FC1021-3 | PuckA_mt1189_A3@15 | 27 | 0.726 | 0.9073 | 0.045793 | 0.000007 | 33.74 | 0.17 |
| FC1021-3 | PuckA_mt1189_A3@16 | 28 | 0.807 | 1.0085 | 0.045676 | 0.000002 | 31.09 | 0.09 |
| FC1021-3 | PuckA_mt1189_A3@17 | 34 | 0.751 | 0.9376 | 0.045426 | 0.000005 | 25.44 | 0.13 |
| FC1021-3 | PuckA_mt1189_A3@18 | 35 | 0.742 | 0.9274 | 0.045663 | 0.000003 | 30.80 | 0.10 |
| FC1021-3 | PuckA_mt1189_A3@19 | 36 | 0.689 | 0.8607 | 0.045588 | 0.000002 | 29.12 | 0.09 |
| FC1021-3 | PuckA_mt1189_A3@20 | 37 | 0.733 | 0.9153 | 0.045755 | 0.000002 | 32.88 | 0.08 |
| FC1020-1A | PuckA_mt1189_A4@22 | 41 | 0.702 | 0.8767 | 0.046374 | 0.000001 | 46.86 | 0.08 |
| FC1020-1A | PuckA_mt1189_A4@23 | 42 | 0.683 | 0.8534 | 0.045821 | 0.000006 | 34.38 | 0.16 |
| FC1020-1A | PuckA_mt1189_A4@24 | 43 | 0.694 | 0.8663 | 0.046468 | 0.000002 | 48.97 | 0.09 |
| FC1020-1A | PuckA_mt1189_A4@25 | 44 | 0.698 | 0.8717 | 0.046408 | 0.000005 | 47.62 | 0.13 |
| FC1020-1B | PuckA_mt1189_A5@26 | 46 | 0.681 | 0.8511 | 0.046203 | 0.000002 | 42.99 | 0.09 |
| FC1020-1B | PuckA_mt1189_A5@27 | 50 | 0.631 | 0.7881 | 0.046447 | 0.000006 | 48.51 | 0.16 |
| FC1020-1B | PuckA_mt1189_A5@28 | 51 | 0.680 | 0.8499 | 0.046114 | 0.000007 | 40.98 | 0.18 |
| FC1020-1B | PuckA_mt1189_A5@29 | 52 | 0.663 | 0.8288 | 0.046121 | 0.000003 | 41.14 | 0.10 |
| FC1020-1B | PuckA_mt1189_A5@30 | 53 | 0.664 | 0.8298 | 0.046353 | 0.000002 | 46.37 | 0.08 |
| FC1020-1B | PuckA_mt1189_A5@31 | 54 | 0.685 | 0.8562 | 0.046285 | 0.000003 | 44.84 | 0.10 |
| FC1020-1B | PuckA_mt1189_A5@32 | 55 | 0.648 | 0.8093 | 0.046547 | 0.000002 | 50.75 | 0.09 |
| FC1020-1B | PuckA_mt1189_A5@33 | 58 | 0.689 | 0.8612 | 0.046415 | 0.000002 | 47.77 | 0.08 |
| FC1020-1B | PuckA_mt1189_A5@35 | 60 | 0.602 | 0.7517 | 0.046266 | 0.000002 | 44.41 | 0.09 |
| FC1020-1B | PuckA_mt1189_A5@36 | 61 | 0.664 | 0.8295 | 0.046188 | 0.000003 | 42.65 | 0.10 |
| FC1020-1B | PuckA_mt1189_A5@38 | 67 | 0.624 | 0.7797 | 0.046368 | 0.000002 | 46.72 | 0.08 |
| FC1020-1B | PuckA_mt1189_A5@39 | 68 | 0.620 | 0.7745 | 0.046560 | 0.000002 | 51.05 | 0.09 |
| FC1020-1B | PuckA_mt1189_A5@40 | 69 | 0.601 | 0.7509 | 0.046410 | 0.000003 | 47.67 | 0.10 |
| FC1020-1B | PuckA_mt1189_A5@41 | 70 | 0.624 | 0.7794 | 0.046419 | 0.000010 | 47.86 | 0.23 |
| FC1020-1B | PuckA_mt1189_A5@43 | 77 | 0.639 | 0.7988 | 0.046330 | 0.000003 | 45.86 | 0.10 |
| FC1020-1B | PuckA_mt1189_A5@44 | 79 | 0.632 | 0.7893 | 0.046411 | 0.000003 | 47.69 | 0.11 |
| **Standards** | | | | | | | | |
| | Bal_sul5A_mt1198_@1 | 5 | 0.947 | 1.1828 | 0.045016 | 0.000001 | 16.20 | 0.08 |
| | Bal_sul5A_mt1198_@2 | 6 | 0.947 | 1.1826 | 0.045018 | 0.000001 | 16.24 | 0.08 |
| | Bal_sul5A_mt1198_@3 | 23 | 0.864 | 1.0788 | 0.045023 | 0.000001 | 16.36 | 0.08 |
| | Bal_sul5A_mt1198_@4 | 32 | 0.807 | 1.0081 | 0.045019 | 0.000001 | 16.26 | 0.08 |
| | Bal_sul5A_mt1198_@5 | 49 | 0.709 | 0.8857 | 0.045016 | 0.000002 | 16.20 | 0.08 |
| | Bal_sul5A_mt1198_@6 | 74 | 0.678 | 0.8472 | 0.045022 | 0.000001 | 16.34 | 0.08 |
| | Rut_sul5A_mt1198_@1 | 1 | 0.987 | | 0.044362 | 0.000001 | 1.44 | 0.07 |
| | Rut_sul5A_mt1198_@02 | 2 | 0.970 | | 0.044361 | 0.000001 | 1.40 | 0.07 |
| | Rut_sul5A_mt1198_@03 | 3 | 0.975 | | 0.044363 | 0.000001 | 1.45 | 0.07 |
| | Rut_sul5A_mt1198_@04 | 4 | 0.976 | | 0.044362 | 0.000001 | 1.44 | 0.07 |
| | Rut_sul5A_mt1198_@05 | 13 | 0.920 | | 0.044360 | 0.000001 | 1.40 | 0.07 |
| | Rut_sul5A_mt1198_@06 | 14 | 0.908 | | 0.044361 | 0.000001 | 1.40 | 0.07 |
| | Rut_sul5A_mt1198_@07 | 21 | 0.872 | | 0.044361 | 0.000001 | 1.41 | 0.08 |
| | Rut_sul5A_mt1198_@08 | 22 | 0.863 | | 0.044360 | 0.000001 | 1.38 | 0.08 |
| | Rut_sul5A_mt1198_@09 | 30 | 0.833 | | 0.044362 | 0.000001 | 1.43 | 0.07 |

| | | | | | | | | |
|---|---|---|---|---|---|---|---|---|
| | Rut_sul5A_mt1198_@10 | 31 | 0.828 | | 0.044360 | 0.000001 | 1.38 | 0.07 |
| | Rut_sul5A_mt1198_@11 | 39 | 0.767 | | 0.044359 | 0.000001 | 1.38 | 0.08 |
| | Rut_sul5A_mt1198_@12 | 40 | 0.750 | | 0.044355 | 0.000001 | 1.29 | 0.07 |
| | Rut_sul5A_mt1198_@13 | 47 | 0.712 | | 0.044361 | 0.000001 | 1.40 | 0.08 |
| | Rut_sul5A_mt1198_@14 | 48 | 0.713 | | 0.044358 | 0.000002 | 1.35 | 0.08 |
| | Rut_sul5A_mt1198_@15 | 56 | 0.733 | | 0.044364 | 0.000002 | 1.47 | 0.08 |
| | Rut_sul5A_mt1198_@16 | 57 | 0.727 | | 0.044363 | 0.000001 | 1.46 | 0.08 |
| | Rut_sul5A_mt1198_@17 | 64 | 0.697 | | 0.044366 | 0.000002 | 1.51 | 0.08 |
| | Rut_sul5A_mt1198_@18 | 65 | 0.698 | | 0.044364 | 0.000001 | 1.47 | 0.08 |
| | Rut_sul5A_mt1198_@19 | 72 | 0.672 | | 0.044352 | 0.000002 | 1.20 | 0.08 |
| | Rut_sul5A_mt1198_@20 | 73 | 0.673 | | 0.044358 | 0.000001 | 1.35 | 0.08 |
| | Rut_sul5A_mt1198_@21 | 80 | 0.667 | | 0.044363 | 0.000002 | 1.46 | 0.08 |
| | Rut_sul5A_mt1198_@22 | 81 | 0.672 | | 0.044365 | 0.000001 | 1.50 | 0.07 |
| Session 2 - Puck B | | | | | | | | |
| FC1020-6a | PuckB_mt1199_A1@1 | 9 | 0.910 | 1.2112 | 0.046265 | 0.000002 | 44.35 | 0.08 |
| FC1020-6a | PuckB_mt1199_A1@2 | 10 | 0.568 | 0.7560 | 0.046183 | 0.000001 | 42.50 | 0.08 |
| FC1020-6a | PuckB_mt1199_A1@3 | 11 | 0.840 | 1.1177 | 0.046153 | 0.000006 | 41.81 | 0.15 |
| FC1020-6a | PuckB_mt1199_A1@5 | 13 | 0.841 | 1.1196 | 0.046132 | 0.000002 | 41.35 | 0.08 |
| FC1020-6a | PuckB_mt1199_A1@7 | 19 | 0.797 | 1.0607 | 0.046575 | 0.000007 | 51.33 | 0.18 |
| FC1020-6a | PuckB_mt1199_A1@8 | 20 | 0.708 | 0.9418 | 0.046843 | 0.000007 | 57.38 | 0.17 |
| FC1020-6a | PuckB_mt1199_A1@9 | 21 | 0.793 | 1.0547 | 0.046200 | 0.000009 | 42.86 | 0.21 |
| FC1020-6b | PuckB_mt1199_A1@12 | 27 | 0.686 | 0.9126 | 0.046360 | 0.000001 | 46.48 | 0.08 |
| FC1020-6b | PuckB_mt1199_A1@13 | 28 | 0.781 | 1.0387 | 0.045017 | 0.000002 | 16.17 | 0.08 |
| FC1020-6b | PuckB_mt1199_A1@14 | 29 | 0.763 | 1.0158 | 0.045928 | 0.000009 | 36.73 | 0.22 |
| FC1020-6b | PuckB_mt1199_A1@15 | 30 | 0.707 | 0.9413 | 0.046097 | 0.000002 | 40.55 | 0.08 |
| FC1020-6b | PuckB_mt1199_A1@18 | 36 | 0.688 | 0.9161 | 0.045154 | 0.000003 | 19.27 | 0.09 |
| **Standards** | | | | | | | | |
| | Bal_sul5A_mt1199_@1 | 5 | 0.707 | 0.9410 | 0.045041 | 0.000002 | 16.72 | 0.08 |
| | Bal_sul5A_mt1199_@2 | 6 | 0.719 | 0.9562 | 0.045037 | 0.000001 | 16.61 | 0.08 |
| | Bal_sul5A_mt1199_@3 | 16 | 0.671 | 0.8933 | 0.045028 | 0.000001 | 16.43 | 0.07 |
| | Bal_sul5A_mt1199_@4 | 17 | 0.679 | 0.9037 | 0.045030 | 0.000001 | 16.47 | 0.07 |
| | Bal_sul5A_mt1199_@5 | 25 | 0.629 | 0.8366 | 0.045036 | 0.000001 | 16.61 | 0.07 |
| | Bal_sul5A_mt1199_@6 | 33 | 0.591 | 0.7871 | 0.045038 | 0.000001 | 16.65 | 0.07 |
| | Bal_sul5A_mt1199_@7 | 39 | 0.506 | 0.6737 | 0.045049 | 0.000002 | 16.89 | 0.08 |
| | Rut_sul5A_mt1199_@1 | 1 | 0.845 | | 0.044362 | 0.000001 | 1.38 | 0.07 |
| | Rut_sul5A_mt1199_@02 | 2 | 0.843 | | 0.044365 | 0.000001 | 1.46 | 0.07 |
| | Rut_sul5A_mt1199_@03 | 3 | 0.811 | | 0.044367 | 0.000001 | 1.51 | 0.07 |
| | Rut_sul5A_mt1199_@04 | 4 | 0.815 | | 0.044364 | 0.000001 | 1.43 | 0.07 |
| | Rut_sul5A_mt1199_@05 | 7 | 0.803 | | 0.044362 | 0.000001 | 1.38 | 0.07 |
| | Rut_sul5A_mt1199_@06 | 8 | 0.796 | | 0.044364 | 0.000001 | 1.44 | 0.07 |
| | Rut_sul5A_mt1199_@07 | 14 | 0.751 | | 0.044359 | 0.000001 | 1.32 | 0.07 |
| | Rut_sul5A_mt1199_@08 | 15 | 0.752 | | 0.044361 | 0.000002 | 1.37 | 0.08 |
| | Rut_sul5A_mt1199_@09 | 23 | 0.730 | | 0.044357 | 0.000001 | 1.27 | 0.07 |
| | Rut_sul5A_mt1199_@10 | 24 | 0.741 | | 0.044363 | 0.000001 | 1.42 | 0.07 |
| | Rut_sul5A_mt1199_@11 | 31 | 0.711 | | 0.044361 | 0.000001 | 1.36 | 0.07 |
| | Rut_sul5A_mt1199_@12 | 32 | 0.662 | | 0.044366 | 0.000001 | 1.48 | 0.07 |
| | Rut_sul5A_mt1199_@13 | 37 | 0.632 | | 0.044363 | 0.000001 | 1.41 | 0.07 |
| | Rut_sul5A_mt1199_@14 | 38 | 0.628 | | 0.044367 | 0.000002 | 1.49 | 0.08 |
| Session 3 - Puck C | | | | | | | | |
| FC1020-7 | PuckC_mt1200@1 | 14 | 0.871 | 2.0413 | 0.046223 | 0.000001 | 42.73 | 0.14 |
| FC1020-7 | PuckC_mt1200@2 | 15 | 0.876 | 2.0532 | 0.046191 | 0.000002 | 42.01 | 0.14 |
| FC1020-7 | PuckC_mt1200@3 | 16 | 0.775 | 1.8148 | 0.046408 | 0.000003 | 46.90 | 0.15 |
| FC1020-7 | PuckC_mt1200@4 | 17 | 0.834 | 1.9546 | 0.046418 | 0.000002 | 47.12 | 0.14 |
| FC1020-7 | PuckC_mt1200@6 | 24 | 0.780 | 1.8279 | 0.045798 | 0.000005 | 33.14 | 0.18 |
| FC1020-7 | PuckC_mt1200@9 | 30 | 0.858 | 2.0091 | 0.046585 | 0.000006 | 50.90 | 0.20 |

| | | | | | | | | |
|---|---|---|---|---|---|---|---|---|
| **Standards** | | | | | | | | |
| | Bal_sul5B_mt1200_@1 | 6 | 0.445 | 1.0425 | 0.045054 | 0.000002 | 16.36 | 0.13 |
| | Bal_sul5B_mt1200_@2 | 7 | 0.439 | 1.0274 | 0.045052 | 0.000003 | 16.32 | 0.14 |
| | Bal_sul5B_mt1200_@3 | 8 | 0.437 | 1.0226 | 0.045051 | 0.000002 | 16.29 | 0.14 |
| | Bal_sul5B_mt1200_@4 | 21 | 0.406 | 0.9512 | 0.045046 | 0.000001 | 16.18 | 0.13 |
| | Bal_sul5B_mt1200_@5 | 22 | 0.409 | 0.9573 | 0.045048 | 0.000002 | 16.22 | 0.13 |
| | Bal_sul5B_mt1200_@6 | 29 | 0.402 | 0.9408 | 0.045049 | 0.000002 | 16.24 | 0.14 |
| | Bal_sul5B_mt1200_@7 | 35 | 0.399 | 0.9349 | 0.045049 | 0.000002 | 16.24 | 0.13 |
| | Bal_sul5B_mt1200_@8 | 36 | 0.394 | 0.9231 | 0.045049 | 0.000002 | 16.25 | 0.14 |
| | Rut_sul5B_mt1200_@1 | 1 | 0.446 | | 0.044383 | 0.000001 | 1.23 | 0.13 |
| | Rut_sul5B_mt1200_@2 | 2 | 0.444 | | 0.044390 | 0.000002 | 1.37 | 0.13 |
| | Rut_sul5B_mt1200_@3 | 3 | 0.442 | | 0.044392 | 0.000001 | 1.43 | 0.13 |
| | Rut_sul5B_mt1200_@4 | 4 | 0.443 | | 0.044393 | 0.000002 | 1.44 | 0.14 |
| | Rut_sul5B_mt1200_@5 | 5 | 0.448 | | 0.044387 | 0.000001 | 1.31 | 0.13 |
| | Rut_sul5B_mt1200_@6 | 9 | 0.437 | | 0.044390 | 0.000002 | 1.38 | 0.13 |
| | Rut_sul5B_mt1200_@7 | 10 | 0.430 | | 0.044394 | 0.000002 | 1.47 | 0.13 |
| | Rut_sul5B_mt1200_@8 | 11 | 0.430 | | 0.044388 | 0.000002 | 1.34 | 0.13 |
| | Rut_sul5B_mt1200_@9 | 12 | 0.432 | | 0.044395 | 0.000002 | 1.49 | 0.13 |
| | Rut_sul5B_mt1200_@10 | 13 | 0.427 | | 0.044396 | 0.000002 | 1.51 | 0.13 |
| | Rut_sul5B_mt1200_@11 | 18 | 0.419 | | 0.044392 | 0.000001 | 1.43 | 0.13 |
| | Rut_sul5B_mt1200_@12 | 19 | 0.416 | | 0.044392 | 0.000002 | 1.42 | 0.13 |
| | Rut_sul5B_mt1200_@13 | 20 | 0.416 | | 0.044397 | 0.000002 | 1.54 | 0.14 |
| | Rut_sul5B_mt1200_@14 | 27 | 0.413 | | 0.044399 | 0.000002 | 1.57 | 0.14 |
| | Rut_sul5B_mt1200_@15 | 28 | 0.417 | | 0.044391 | 0.000002 | 1.40 | 0.13 |
| | Rut_sul5B_mt1200_@16 | 33 | 0.425 | | 0.044401 | 0.000002 | 1.63 | 0.13 |
| | Rut_sul5B_mt1200_@17 | 34 | 0.431 | | 0.044393 | 0.000002 | 1.45 | 0.13 |
| | Rut_sul5B_mt1200_@18 | 37 | 0.396 | | 0.044378 | 0.000001 | 1.10 | 0.13 |
| | Rut_sul5B_mt1200_@19 | 38 | 0.401 | | 0.044384 | 0.000002 | 1.25 | 0.14 |
| Session 4 - Puck D | | | | | | | | |
| FC1020-4 | PuckD_mt1201_D1@1 | 11 | 0.900 | 1.1219 | 0.046724 | 0.000004 | 54.63 | 0.10 |
| FC1020-4 | PuckD_mt1201_D1@2 | 12 | 0.860 | 1.0711 | 0.046051 | 0.000002 | 39.46 | 0.08 |
| FC1020-4 | PuckD_mt1201_D1@4 | 14 | 0.879 | 1.0953 | 0.046568 | 0.000002 | 51.13 | 0.07 |
| FC1020-4 | PuckD_mt1201_D1@5 | 15 | 0.879 | 1.0955 | 0.046555 | 0.000003 | 50.82 | 0.08 |
| FC1020-4 | PuckD_mt1201_D1@6 | 16 | 0.879 | 1.0957 | 0.046742 | 0.000008 | 55.06 | 0.20 |
| FC1020-4 | PuckD_mt1201_D1@7 | 17 | 0.888 | 1.1066 | 0.046856 | 0.000004 | 57.62 | 0.11 |
| FC1020-4 | PuckD_mt1201_D1@8 | 18 | 0.880 | 1.0968 | 0.046257 | 0.000006 | 44.11 | 0.14 |
| FC1020-4 | PuckD_mt1201_D1@10 | 26 | 0.842 | 1.0490 | 0.046280 | 0.000002 | 44.61 | 0.07 |
| FC1020-4 | PuckD_mt1201_D1@11 | 27 | 0.829 | 1.0328 | 0.046560 | 0.000001 | 50.94 | 0.06 |
| FC1020-4 | PuckD_mt1201_D1@12 | 28 | 0.831 | 1.0358 | 0.046401 | 0.000009 | 47.35 | 0.21 |
| FC1020-4 | PuckD_mt1201_D1@13 | 29 | 0.872 | 1.0870 | 0.045467 | 0.000001 | 26.28 | 0.06 |
| FC1020-4 | PuckD_mt1201_D1@14 | 30 | 0.827 | 1.0303 | 0.046145 | 0.000009 | 41.57 | 0.20 |
| FC1020-4 | PuckD_mt1201_D1@15 | 31 | 0.831 | 1.0353 | 0.046836 | 0.000002 | 57.17 | 0.08 |
| FC1020-4 | PuckD_mt1201_D1@16 | 32 | 0.835 | 1.0409 | 0.046294 | 0.000002 | 44.93 | 0.07 |
| FC1020-4 | PuckD_mt1201_D1@17 | 33 | 0.840 | 1.0461 | 0.046550 | 0.000002 | 50.72 | 0.07 |
| FC1020-4 | PuckD_mt1201_D2@18 | 42 | 0.804 | 1.0017 | 0.046825 | 0.000006 | 56.93 | 0.14 |
| FC1020-4 | PuckD_mt1201_D2@19 | 43 | 0.823 | 1.0256 | 0.046509 | 0.000009 | 49.80 | 0.22 |
| FC1020-4 | PuckD_mt1201_D2@20 | 44 | 0.814 | 1.0142 | 0.046407 | 0.000002 | 47.50 | 0.07 |
| FC1020-4 | PuckD_mt1201_D2@21 | 45 | 0.817 | 1.0180 | 0.046738 | 0.000003 | 54.97 | 0.08 |
| FC1020-4 | PuckD_mt1201_D2@22 | 46 | 0.810 | 1.0094 | 0.046457 | 0.000004 | 48.62 | 0.10 |
| FC1020-4 | PuckD_mt1201_D3@23 | 47 | 0.827 | 1.0302 | 0.046419 | 0.000002 | 47.77 | 0.07 |
| FC1020-4 | PuckD_mt1201_D3@24 | 48 | 0.802 | 0.9996 | 0.045931 | 0.000001 | 36.73 | 0.06 |
| FC1020-4 | PuckD_mt1201_D3@25 | 49 | 0.807 | 1.0049 | 0.046411 | 0.000003 | 47.58 | 0.08 |
| **Standards** | | | | | | | | |
| | Bal_sul5B_mt1201_@1 | 5 | 0.832 | 1.0364 | 0.045029 | 0.000001 | 16.38 | 0.06 |
| | Bal_sul5B_mt1201_@2 | 6 | 0.837 | 1.0434 | 0.045027 | 0.000001 | 16.33 | 0.06 |
| | Bal_sul5B_mt1201_@3 | 25 | 0.788 | 0.9811 | 0.045029 | 0.000002 | 16.37 | 0.06 |
| | Bal_sul5B_mt1201_@4 | 40 | 0.778 | 0.9696 | 0.045027 | 0.000002 | 16.34 | 0.07 |
| | Bal_sul5B_mt1201_@5 | 41 | 0.786 | 0.9794 | 0.045027 | 0.000001 | 16.33 | 0.06 |

| | | | | | | | |
|---|---|---|---|---|---|---|---|
| Bal_sul5B_mt1201_@6 | 56 | 0.752 | 0.9364 | 0.045021 | 0.000001 | 16.19 | 0.06 |
| Bal_sul5B_mt1201_@7 | 57 | 0.758 | 0.9441 | 0.045022 | 0.000001 | 16.24 | 0.06 |
| Rut_sul5B_mt1201_@1 | 1 | 0.811 | | 0.044366 | 0.000001 | 1.41 | 0.06 |
| Rut_sul5B_mt1201_@02 | 2 | 0.828 | | 0.044365 | 0.000001 | 1.40 | 0.06 |
| Rut_sul5B_mt1201_@03 | 3 | 0.840 | | 0.044368 | 0.000001 | 1.46 | 0.06 |
| Rut_sul5B_mt1201_@04 | 4 | 0.812 | | 0.044368 | 0.000001 | 1.46 | 0.06 |
| Rut_sul5B_mt1201_@05 | 7 | 0.832 | | 0.044365 | 0.000001 | 1.39 | 0.06 |
| Rut_sul5B_mt1201_@06 | 8 | 0.810 | | 0.044363 | 0.000001 | 1.36 | 0.06 |
| Rut_sul5B_mt1201_@07 | 9 | 0.824 | | 0.044361 | 0.000001 | 1.32 | 0.06 |
| Rut_sul5B_mt1201_@08 | 10 | 0.820 | | 0.044366 | 0.000001 | 1.41 | 0.06 |
| Rut_sul5B_mt1201_@09 | 21 | 0.798 | | 0.044367 | 0.000001 | 1.44 | 0.06 |
| Rut_sul5B_mt1201_@10 | 22 | 0.808 | | 0.044366 | 0.000001 | 1.42 | 0.06 |
| Rut_sul5B_mt1201_@11 | 23 | 0.823 | | 0.044366 | 0.000001 | 1.43 | 0.06 |
| Rut_sul5B_mt1201_@12 | 24 | 0.828 | | 0.044366 | 0.000001 | 1.43 | 0.06 |
| Rut_sul5B_mt1201_@13 | 36 | 0.814 | | 0.044365 | 0.000001 | 1.39 | 0.06 |
| Rut_sul5B_mt1201_@14 | 37 | 0.831 | | 0.044363 | 0.000001 | 1.34 | 0.06 |
| Rut_sul5B_mt1201_@15 | 38 | 0.768 | | 0.044364 | 0.000002 | 1.38 | 0.07 |
| Rut_sul5B_mt1201_@16 | 39 | 0.776 | | 0.044368 | 0.000001 | 1.46 | 0.06 |
| Rut_sul5B_mt1201_@17 | 52 | 0.747 | | 0.044371 | 0.000001 | 1.53 | 0.06 |
| Rut_sul5B_mt1201_@18 | 53 | 0.755 | | 0.044366 | 0.000001 | 1.43 | 0.06 |
| Rut_sul5B_mt1201_@19 | 54 | 0.759 | | 0.044365 | 0.000001 | 1.39 | 0.06 |
| Rut_sul5B_mt1201_@20 | 55 | 0.769 | | 0.044361 | 0.000001 | 1.31 | 0.06 |

**Table 2. Results of the ion microprobe iron isotope analyses**

| Sample name | Sample ID | Seq. in run | 56Fe cps (x 109) | 56Fesamp/av.std | 56Fe/54Fe drift corrected | ± abs | δ56Fe samples | ± ‰ |
|---|---|---|---|---|---|---|---|---|
| **Session 5 - Puck A** | | | | | | | | |
| FC1021-2 | PuckA_mt1198_A1@01 | 4 | 0.190 | 0.9404 | 15.164815 | 0.000939 | -0.68 | 0.10 |
| FC1021-2 | PuckA_mt1198_A1@02 | 5 | 0.189 | 0.9387 | 15.167160 | 0.000676 | -0.53 | 0.09 |
| FC1021-2 | PuckA_mt1198_A1@03 | 6 | 0.190 | 0.9432 | 15.168315 | 0.000543 | -0.45 | 0.09 |
| FC1021-2 | PuckA_mt1198_A1@04 | 7 | 0.190 | 0.9426 | 15.167540 | 0.000779 | -0.50 | 0.10 |
| FC1021-2 | PuckA_mt1198_A1@05 | 8 | 0.190 | 0.9416 | 15.167305 | 0.000544 | -0.52 | 0.09 |
| FC1021-2 | PuckA_mt1198_A1@06 | 9 | 0.190 | 0.9417 | 15.172850 | 0.000984 | -0.15 | 0.11 |
| FC1021-1 | PuckA_mt1198_A2@07 | 12 | 0.181 | 0.8989 | 15.167594 | 0.000619 | -0.50 | 0.09 |
| FC1021-1 | PuckA_mt1198_A2@08 | 13 | 0.182 | 0.9046 | 15.167099 | 0.000687 | -0.53 | 0.09 |
| FC1021-1 | PuckA_mt1198_A2@09 | 14 | 0.180 | 0.8951 | 15.171154 | 0.000558 | -0.27 | 0.09 |
| FC1021-1 | PuckA_mt1198_A2@10 | 15 | 0.180 | 0.8910 | 15.169139 | 0.001017 | -0.40 | 0.11 |
| FC1021-1 | PuckA_mt1198_A2@11 | 16 | 0.181 | 0.8963 | 15.169084 | 0.000693 | -0.40 | 0.09 |
| FC1021-1 | PuckA_mt1198_A2@12 | 17 | 0.184 | 0.9121 | 15.161239 | 0.000643 | -0.92 | 0.09 |
| FC1021-3 | PuckA_mt1198_A3@13 | 22 | 0.174 | 0.8649 | 15.167794 | 0.000689 | -0.49 | 0.09 |
| FC1021-3 | PuckA_mt1198_A3@14 | 23 | 0.174 | 0.8636 | 15.163229 | 0.000593 | -0.79 | 0.09 |
| FC1021-3 | PuckA_mt1198_A3@15 | 24 | 0.174 | 0.8637 | 15.163584 | 0.000649 | -0.76 | 0.09 |
| FC1021-3 | PuckA_mt1198_A3@16 | 25 | 0.174 | 0.8617 | 15.162009 | 0.000576 | -0.87 | 0.09 |
| FC1021-3 | PuckA_mt1198_A3@17 | 29 | 0.176 | 0.8713 | 15.164858 | 0.000565 | -0.68 | 0.09 |
| FC1021-3 | PuckA_mt1198_A3@18 | 30 | 0.175 | 0.8684 | 15.174003 | 0.000982 | -0.08 | 0.11 |
| FC1021-3 | PuckA_mt1198_A3@19 | 31 | 0.176 | 0.8717 | 15.165578 | 0.000692 | -0.63 | 0.09 |
| FC1020-1A | PuckA_mt1198_A4@21 | 33 | 0.173 | 0.8573 | 15.169338 | 0.001292 | -0.38 | 0.12 |
| FC1020-1A | PuckA_mt1198_A4@22 | 34 | 0.175 | 0.8694 | 15.164173 | 0.000678 | -0.73 | 0.09 |
| FC1020-1A | PuckA_mt1198_A4@23 | 38 | 0.170 | 0.8441 | 15.177653 | 0.001002 | 0.16 | 0.11 |
| FC1020-1A | PuckA_mt1198_A4@24 | 39 | 0.172 | 0.8525 | 15.166868 | 0.000779 | -0.55 | 0.10 |
| FC1020-1A | PuckA_mt1198_A4@25 | 40 | 0.171 | 0.8509 | 15.162253 | 0.000667 | -0.85 | 0.09 |
| FC1020-1B | PuckA_mt1198_A5@26 | 42 | 0.171 | 0.8474 | 15.161843 | 0.000573 | -0.88 | 0.09 |
| FC1020-1B | PuckA_mt1198_A5@27 | 43 | 0.173 | 0.8599 | 15.163038 | 0.000738 | -0.80 | 0.10 |
| FC1020-1B | PuckA_mt1198_A5@28 | 46 | 0.171 | 0.8489 | 15.174352 | 0.000680 | -0.05 | 0.09 |
| FC1020-1B | PuckA_mt1198_A5@29 | 47 | 0.173 | 0.8575 | 15.167317 | 0.000600 | -0.52 | 0.09 |
| FC1020-1B | PuckA_mt1198_A5@31 | 49 | 0.174 | 0.8647 | 15.165467 | 0.000753 | -0.64 | 0.10 |
| FC1020-1B | PuckA_mt1198_A5@32 | 50 | 0.171 | 0.8467 | 15.159932 | 0.000602 | -1.00 | 0.09 |
| FC1020-1B | PuckA_mt1198_A5@33 | 51 | 0.170 | 0.8442 | 15.175377 | 0.000965 | 0.01 | 0.10 |
| FC1020-1B | PuckA_mt1198_A5@35 | 56 | 0.170 | 0.8427 | 15.165652 | 0.000785 | -0.63 | 0.10 |

| | | | | | | | | |
|---|---|---|---|---|---|---|---|---|
| FC1020-1B | PuckA_mt1198_A5@36 | 57 | 0.171 | 0.8473 | 15.163707 | 0.000609 | -0.76 | 0.09 |
| FC1020-1B | PuckA_mt1198_A5@37 | 58 | 0.171 | 0.8480 | 15.162962 | 0.000837 | -0.81 | 0.10 |
| FC1020-1B | PuckA_mt1198_A5@38 | 60 | 0.175 | 0.8700 | 15.172092 | 0.000690 | -0.20 | 0.09 |
| FC1020-1B | PuckA_mt1198_A5@43 | 64 | 0.167 | 0.8273 | 15.168211 | 0.000701 | -0.46 | 0.10 |
| **Standards** | | | | | | | | |
| | Bal-Fe_sul5B_mt1198_@1 | 1 | 0.202 | | 15.168340 | 0.000626 | -0.45 | 0.09 |
| | Bal-Fe_sul5B_mt1198_@2 | 2 | 0.201 | | 15.169015 | 0.000663 | -0.41 | 0.09 |
| | Bal-Fe_sul5B_mt1198_@3 | 3 | 0.202 | | 15.168220 | 0.000590 | -0.46 | 0.09 |
| | Bal-Fe_sul5B_mt1198_@4 | 10 | 0.204 | | 15.169674 | 0.000757 | -0.36 | 0.10 |
| | Bal-Fe_sul5B_mt1198_@5 | 11 | 0.204 | | 15.170119 | 0.000951 | -0.33 | 0.10 |
| | Bal-Fe_sul5B_mt1198_@6 | 19 | 0.204 | | 15.168449 | 0.000524 | -0.44 | 0.09 |
| | Bal-Fe_sul5B_mt1198_@7 | 20 | 0.206 | | 15.168184 | 0.000729 | -0.46 | 0.10 |
| | Bal-Fe_sul5B_mt1198_@8 | 27 | 0.198 | | 15.170159 | 0.000733 | -0.33 | 0.10 |
| | Bal-Fe_sul5B_mt1198_@9 | 28 | 0.200 | | 15.172173 | 0.000624 | -0.20 | 0.09 |
| | Bal-Fe_sul5B_mt1198_@10 | 36 | 0.198 | | 15.167713 | 0.000532 | -0.49 | 0.09 |
| | Bal-Fe_sul5B_mt1198_@11 | 37 | 0.201 | | 15.171048 | 0.000770 | -0.27 | 0.10 |
| | Bal-Fe_sul5B_mt1198_@12 | 44 | 0.203 | | 15.167593 | 0.000588 | -0.50 | 0.09 |
| | Bal-Fe_sul5B_mt1198_@13 | 45 | 0.206 | | 15.169208 | 0.000710 | -0.39 | 0.10 |
| | Bal-Fe_sul5B_mt1198_@14 | 53 | 0.203 | | 15.168577 | 0.000634 | -0.44 | 0.09 |
| | Bal-Fe_sul5B_mt1198_@15 | 54 | 0.203 | | 15.167282 | 0.000650 | -0.52 | 0.09 |
| | Bal-Fe_sul5B_mt1198_@16 | 61 | 0.203 | | 15.168607 | 0.000639 | -0.43 | 0.09 |
| | Bal-Fe_sul5B_mt1198_@17 | 62 | 0.194 | | 15.169572 | 0.000808 | -0.37 | 0.10 |
| | Bal-Fe_sul5B_mt1198_@18 | 69 | 0.197 | | 15.170496 | 0.000827 | -0.31 | 0.10 |
| | Bal-Fe_sul5B_mt1198_@19 | 70 | 0.198 | | 15.168931 | 0.000716 | -0.41 | 0.10 |
| | Isu-Fe_sul5B_mt1198_@1 | 18 | 0.206 | 1.0208 | 15.189114 | 0.000524 | 0.92 | 0.09 |
| | Isu-Fe_sul5B_mt1198_@2 | 35 | 0.203 | 1.0073 | 15.188218 | 0.000589 | 0.86 | 0.09 |
| | Isu-Fe_sul5B_mt1198_@3 | 52 | 0.204 | 1.0118 | 15.189612 | 0.000675 | 0.95 | 0.09 |
| | Isu-Fe_sul5B_mt1198_@4 | 67 | 0.202 | 1.0018 | 15.188306 | 0.000625 | 0.87 | 0.09 |
| | Isu-Fe_sul5B_mt1198_@5 | 68 | 0.204 | 1.0126 | 15.188231 | 0.000539 | 0.86 | 0.09 |
| Session 6 - Puck B | | | | | | | | |
| FC1020-6a | PuckB_mt1199_@01 | 5 | 0.221 | 0.9077 | 15.136333 | 0.000700 | 0.44 | 0.20 |
| FC1020-6a | PuckB_mt1199_@02 | 6 | 0.215 | 0.8826 | 15.131269 | 0.000527 | 0.10 | 0.20 |
| FC1020-6a | PuckB_mt1199_@03 | 7 | 0.219 | 0.8990 | 15.133134 | 0.000505 | 0.22 | 0.20 |
| FC1020-6a | PuckB_mt1199_@04 | 8 | 0.217 | 0.8923 | 15.128640 | 0.000507 | -0.07 | 0.20 |
| FC1020-6a | PuckB_mt1199_@05 | 9 | 0.215 | 0.8831 | 15.127806 | 0.000794 | -0.13 | 0.20 |
| FC1020-6a | PuckB_mt1199_@06 | 10 | 0.216 | 0.8848 | 15.139062 | 0.001131 | 0.62 | 0.21 |
| FC1020-6a | PuckB_mt1199_@07 | 13 | 0.217 | 0.8910 | 15.146009 | 0.000668 | 1.08 | 0.20 |
| FC1020-6a | PuckB_mt1199_@08 | 14 | 0.220 | 0.9028 | 15.139934 | 0.000520 | 0.67 | 0.20 |
| FC1020-6a | PuckB_mt1199_@09 | 15 | 0.216 | 0.8873 | 15.143770 | 0.000870 | 0.93 | 0.21 |
| FC1020-6b | PuckB_mt1199_@11 | 17 | 0.201 | 0.8243 | 15.159962 | 0.000724 | 2.00 | 0.20 |
| FC1020-6b | PuckB_mt1199_@13 | 21 | 0.223 | 0.9171 | 15.150755 | 0.000705 | 1.39 | 0.20 |
| FC1020-6b | PuckB_mt1199_@14 | 22 | 0.217 | 0.8926 | 15.149620 | 0.000844 | 1.31 | 0.21 |
| FC1020-6b | PuckB_mt1199_@15 | 23 | 0.217 | 0.8906 | 15.160686 | 0.000618 | 2.05 | 0.20 |
| FC1020-6b | PuckB_mt1199_@16 | 24 | 0.206 | 0.8473 | 15.162702 | 0.000917 | 2.18 | 0.21 |
| FC1020-6b | PuckB_mt1199_@17 | 25 | 0.219 | 0.9001 | 15.162247 | 0.000771 | 2.15 | 0.20 |
| **Standards** | | | | | | | | |
| | Bal-Fe_sul5B_mt1199_@4 | 4 | 0.241 | | 15.120097 | 0.000651 | -0.64 | 0.20 |
| | Bal-Fe_sul5B_mt1199_@5 | 11 | 0.244 | | 15.123967 | 0.000731 | -0.38 | 0.20 |
| | Bal-Fe_sul5B_mt1199_@6 | 12 | 0.246 | | 15.127593 | 0.000603 | -0.14 | 0.20 |
| | Bal-Fe_sul5B_mt1199_@7 | 19 | 0.245 | | 15.127453 | 0.000479 | -0.15 | 0.20 |
| | Bal-Fe_sul5B_mt1199_@8 | 20 | 0.242 | | 15.122319 | 0.000856 | -0.49 | 0.21 |
| | Bal-Fe_sul5B_mt1199_@9 | 27 | 0.243 | | 15.120639 | 0.000538 | -0.60 | 0.20 |
| | Bal-Fe_sul5B_mt1199_@10 | 28 | 0.245 | | 15.123835 | 0.000504 | -0.39 | 0.20 |
| Session 7 - Puck C | | | | | | | | |
| FC1020-7 | PuckC_mt1200_@x1 | 4 | 0.175 | 0.9659 | 15.171580 | 0.000886 | 0.50 | 0.11 |
| FC1020-7 | PuckC_mt1200_@x2 | 5 | 0.173 | 0.9573 | 15.177230 | 0.000861 | 0.87 | 0.11 |

| | | | | | | | | |
|---|---|---|---|---|---|---|---|---|
| FC1020-7 | PuckC_mt1200_@x3 | 6 | 0.178 | 0.9816 | 15.171180 | 0.000960 | 0.47 | 0.11 |
| FC1020-7 | PuckC_mt1200_@x4 | 7 | 0.182 | 1.0021 | 15.166590 | 0.000573 | 0.17 | 0.10 |
| FC1020-7 | PuckC_mt1200_@x9 | 8 | 0.176 | 0.9709 | 15.181410 | 0.000797 | 1.15 | 0.11 |
| FC1020-7 | PuckC_mt1200_@x11 | 9 | 0.170 | 0.9400 | 15.168990 | 0.000574 | 0.33 | 0.10 |
| **Standards** | | | | | | | | |
| | Bal-Fe_sul5B_mt1200_@01 | 1 | 0.181 | | 15.157220 | 0.000767 | -0.45 | 0.11 |
| | Bal-Fe_sul5B_mt1200_@2 | 2 | 0.182 | | 15.159450 | 0.000834 | -0.30 | 0.11 |
| | Bal-Fe_sul5B_mt1200_@3 | 3 | 0.183 | | 15.158790 | 0.000563 | -0.35 | 0.10 |
| | Bal-Fe_sul5B_mt1200_@4 | 10 | 0.179 | | 15.155650 | 0.000763 | -0.55 | 0.11 |
| | Bal-Fe_sul5B_mt1200_@5 | 11 | 0.180 | | 15.159100 | 0.000699 | -0.33 | 0.10 |
| | Bal-Fe_sul5B_mt1200_@6 | 12 | 0.182 | | 15.157670 | 0.000555 | -0.42 | 0.10 |
| Session 8 - Puck D | | | | | | | | |
| FC1020-4 | PuckD_mt1201_1-a | 5 | 0.222 | 1.0648 | 15.178401 | 0.000503 | -0.02 | 0.08 |
| FC1020-4 | PuckD_mt1201_1-b | 6 | 0.220 | 1.0548 | 15.176651 | 0.000622 | -0.13 | 0.09 |
| FC1020-4 | PuckD_mt1201_1-c | 7 | 0.219 | 1.0519 | 15.176332 | 0.000722 | -0.16 | 0.09 |
| FC1020-4 | PuckD_mt1201_1-d | 8 | 0.221 | 1.0618 | 15.178762 | 0.000565 | 0.00 | 0.08 |
| FC1020-4 | PuckD_mt1201_1@x1 | 9 | 0.218 | 1.0446 | 15.176502 | 0.000522 | -0.14 | 0.08 |
| FC1020-4 | PuckD_mt1201_1@x2 | 10 | 0.217 | 1.0419 | 15.192992 | 0.000777 | 0.94 | 0.09 |
| FC1020-4 | PuckD_mt1201_1@x3 | 11 | 0.217 | 1.0416 | 15.183953 | 0.000637 | 0.35 | 0.09 |
| FC1020-4 | PuckD_mt1201_1@x6 | 12 | 0.218 | 1.0459 | 15.178763 | 0.000722 | 0.00 | 0.09 |
| FC1020-4 | PuckD_mt1201_1@x7 | 13 | 0.214 | 1.0238 | 15.187213 | 0.000517 | 0.56 | 0.08 |
| FC1020-4 | PuckD_mt1201_1@x8 | 14 | 0.218 | 1.0440 | 15.182494 | 0.000750 | 0.25 | 0.09 |
| FC1020-4 | PuckD_mt1201_1@x10 | 18 | 0.215 | 1.0289 | 15.177075 | 0.000512 | -0.11 | 0.08 |
| FC1020-4 | PuckD_mt1201_1@x12 | 20 | 0.215 | 1.0310 | 15.179375 | 0.000697 | 0.05 | 0.09 |
| FC1020-4 | PuckD_mt1201_1@x13 | 21 | 0.185 | 0.8857 | 15.188536 | 0.001158 | 0.65 | 0.11 |
| FC1020-4 | PuckD_mt1201_1@x14 | 22 | 0.214 | 1.0278 | 15.180036 | 0.000787 | 0.09 | 0.09 |
| FC1020-4 | PuckD_mt1201_1@x15 | 23 | 0.214 | 1.0244 | 15.175586 | 0.000603 | -0.20 | 0.08 |
| FC1020-4 | PuckD_mt1201_1@x17 | 24 | 0.216 | 1.0349 | 15.183186 | 0.000759 | 0.30 | 0.09 |
| FC1020-4 | PuckD_mt1201_1@x18 | 25 | 0.218 | 1.0433 | 15.178837 | 0.000654 | 0.01 | 0.09 |
| FC1020-4 | PuckD_mt1201_2@x18 | 28 | 0.209 | 1.0044 | 15.173477 | 0.000798 | -0.34 | 0.09 |
| FC1020-4 | PuckD_mt1201_2@x19 | 29 | 0.199 | 0.9553 | 15.178958 | 0.000831 | 0.02 | 0.09 |
| FC1020-4 | PuckD_mt1201_2@x20 | 30 | 0.211 | 1.0103 | 15.176168 | 0.000516 | -0.17 | 0.08 |
| FC1020-4 | PuckD_mt1201_2@x21 | 31 | 0.207 | 0.9900 | 15.184638 | 0.000750 | 0.39 | 0.09 |
| FC1020-4 | PuckD_mt1201_2@x22 | 32 | 0.209 | 1.0016 | 15.180149 | 0.000621 | 0.10 | 0.09 |
| FC1020-4 | PuckD_mt1201_2@x23 | 33 | 0.207 | 0.9925 | 15.181809 | 0.000644 | 0.21 | 0.09 |
| FC1020-4 | PuckD_mt1201_2@x24 | 34 | 0.205 | 0.9848 | 15.174819 | 0.000848 | -0.25 | 0.09 |
| FC1020-4 | PuckD_mt1201_2@x25 | 35 | 0.207 | 0.9940 | 15.180909 | 0.000722 | 0.15 | 0.09 |
| **Standards** | | | | | | | | |
| | Bal-Fe_sul5A_mt1201_test@15 | 1 | 0.204 | | 15.172840 | 0.000525 | -0.39 | 0.08 |
| | Bal-Fe_sul5A_mt1201_test@16 | 2 | 0.208 | | 15.173290 | 0.000631 | -0.36 | 0.09 |
| | Bal-Fe_sul5A_mt1201_test@17 | 3 | 0.213 | | 15.170021 | 0.000771 | -0.57 | 0.09 |
| | Bal-Fe_sul5A_mt1201_test@18 | 4 | 0.212 | | 15.173621 | 0.000515 | -0.33 | 0.08 |
| | Bal-Fe_sul5A_mt1201_test@19 | 15 | 0.204 | | 15.174174 | 0.000773 | -0.30 | 0.09 |
| | Bal-Fe_sul5A_mt1201_test@20 | 16 | 0.208 | | 15.172294 | 0.000520 | -0.42 | 0.08 |
| | Bal-Fe_sul5A_mt1201_test@21 | 17 | 0.210 | | 15.171904 | 0.000517 | -0.45 | 0.08 |
| | Bal-Fe_sul5A_mt1201_test@22 | 26 | 0.210 | | 15.173377 | 0.000555 | -0.35 | 0.08 |
| | Bal-Fe_sul5A_mt1201_test@23 | 27 | 0.209 | | 15.173227 | 0.000598 | -0.36 | 0.08 |
| | Bal-Fe_sul5A_mt1201_test@24 | 36 | 0.209 | | 15.171960 | 0.000576 | -0.44 | 0.08 |
| | Bal-Fe_sul5A_mt1201_test@25 | 37 | 0.209 | | 15.172250 | 0.000777 | -0.42 | 0.09 |

**Table 3 - Results of stable sulfur and iron whole-rock isotope analyses**

| Sample | d34S‰ CDT | 1sd | d57Fe‰ IRMM-14 | 1sd | d56Fe‰ IRMM-14 | 1sd |
|---|---|---|---|---|---|---|
| FC1022 | 17.2 | 0.19 | -0.052 | 0.12 | -0.10 | 0.02 |
| FC1020 | 41.7 | 0.15 | -0.300 | 0.12 | -0.12 | 0.08 |
| FC1019 | 30.3 | 0.15 | -0.369 | 0.14 | -0.48 | 0.46 |
| FC1018 | 28.9 | 0.46 | -0.475 | 0.42 | -0.31 | 0.21 |
| FC1012 | 39.4 | 0.23 | -0.091 | 0.06 | -0.06 | 0.11 |
| FC0915 | 18.1 | 0.20 | -0.367 | 0.06 | -0.50 | 0.18 |
| FC1201 | 30.6 | 0.28 | -0.001 | 0.09 | -0.13 | 0.11 |
| FC10XXX | 24.3 | 0.22 | -0.298 | 0.04 | -0.19 | 0.01 |
| FC09A5b | 33.0 | 0.22 | -0.206 | 0.11 | -0.09 | 0.13 |
| FC09A2b | 13.3 | 0.41 | -0.108 | 0.07 | -0.01 | 0.15 |

**Table 4 - Results of stable sulfur and iron whole-rock isotope analyses - standards**

| Standard | d34S‰ CDT | 1s | average | 1s |
|---|---|---|---|---|
| S3-10ppm-1 | -31.92 | 1.18 | | |
| S3-10ppm-1 | -31.76 | 0.52 | | |
| S3-10ppm-1 | -32.44 | 0.56 | | |
| S3-10ppm-1 | -32.62 | 0.51 | -32.18 | 0.41 |

| Standard | d57Fe | 2SE | d56Fe | 2SE | d57Fe average | 1sd | d56Fe average | 1sd |
|---|---|---|---|---|---|---|---|---|
| Fe-BCR-1 | 0.027 | 0.109 | -0.052 | 0.068 | | | | |
| Fe-BCR-1 | -0.023 | 0.085 | -0.058 | 0.044 | | | | |
| Fe-BCR-1 | -0.076 | 0.090 | 0.042 | 0.065 | | | | |
| Fe-BCR-1 | -0.123 | 0.077 | -0.054 | 0.038 | | | | |
| Fe-BCR-1 | -0.030 | 0.091 | 0.064 | 0.074 | | | | |
| Fe-BCR-1 | -0.170 | 0.093 | -0.042 | 0.062 | | | | |
| Fe-BCR-1 | -0.016 | 0.155 | 0.068 | 0.057 | | | | |
| Fe-BCR-1 | -0.095 | 0.087 | -0.078 | 0.058 | | | | |
| Fe-BCR-1 | 0.449 | 0.737 | 0.250 | 0.335 | -0.007 | 0.181 | 0.016 | 0.105 |
| Fe-BHVO-2 | 0.264 | 0.106 | 0.192 | 0.045 | | | | |
| Fe-BHVO-2 | 0.160 | 0.104 | 0.012 | 0.070 | | | | |
| Fe-BHVO-2 | -0.027 | 0.098 | -0.113 | 0.063 | | | | |
| Fe-BHVO-2 | 0.200 | 0.110 | 0.082 | 0.106 | | | | |
| Fe-BHVO-2 | 0.039 | 0.098 | 0.112 | 0.061 | | | | |
| Fe-BHVO-2 | 0.030 | 0.103 | 0.080 | 0.067 | 0.111 | 0.114 | 0.061 | 0.103 |

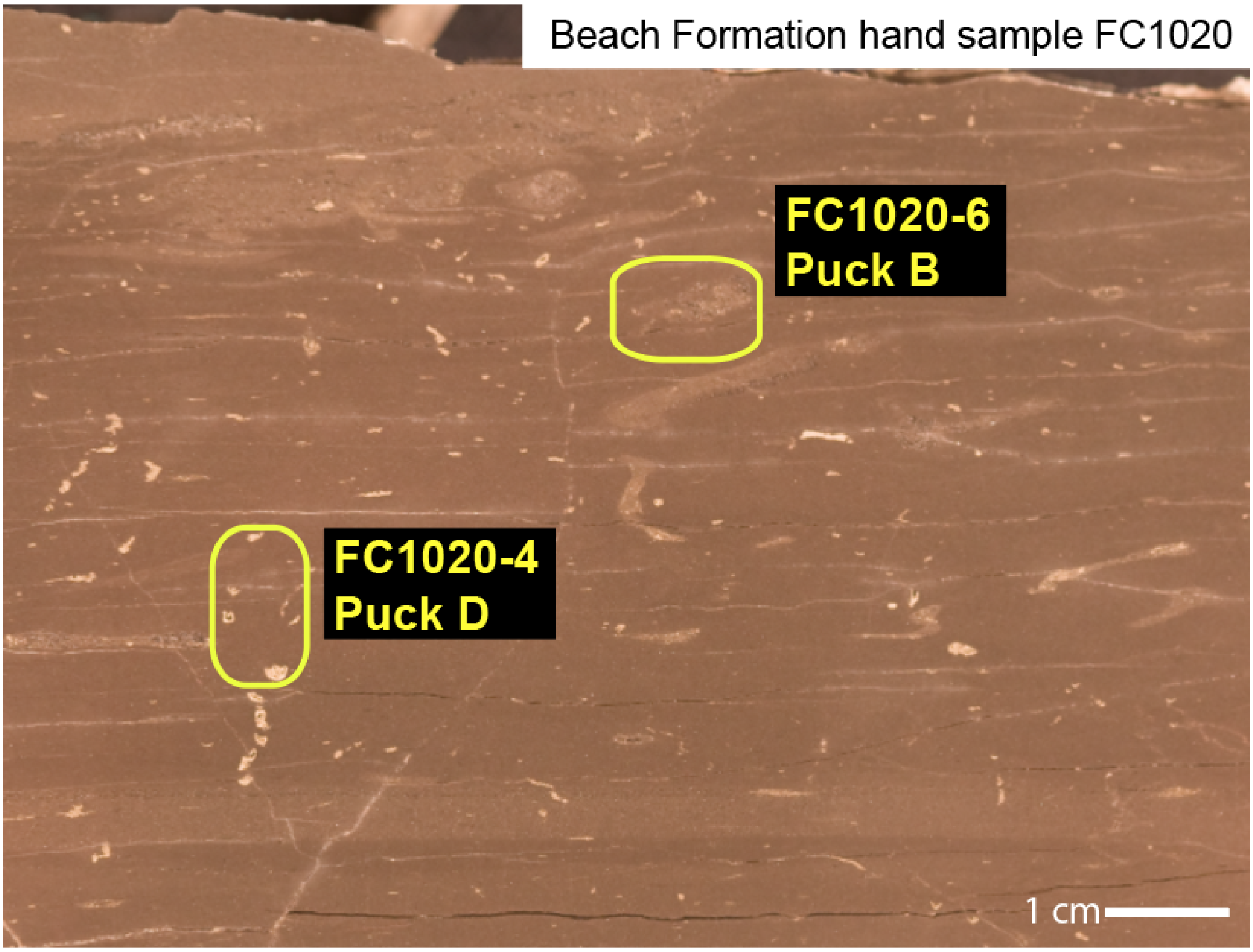
Beach Formation hand sample FC1020
FC1020-6
Puck B
FC1020-4
Puck D
1 cm

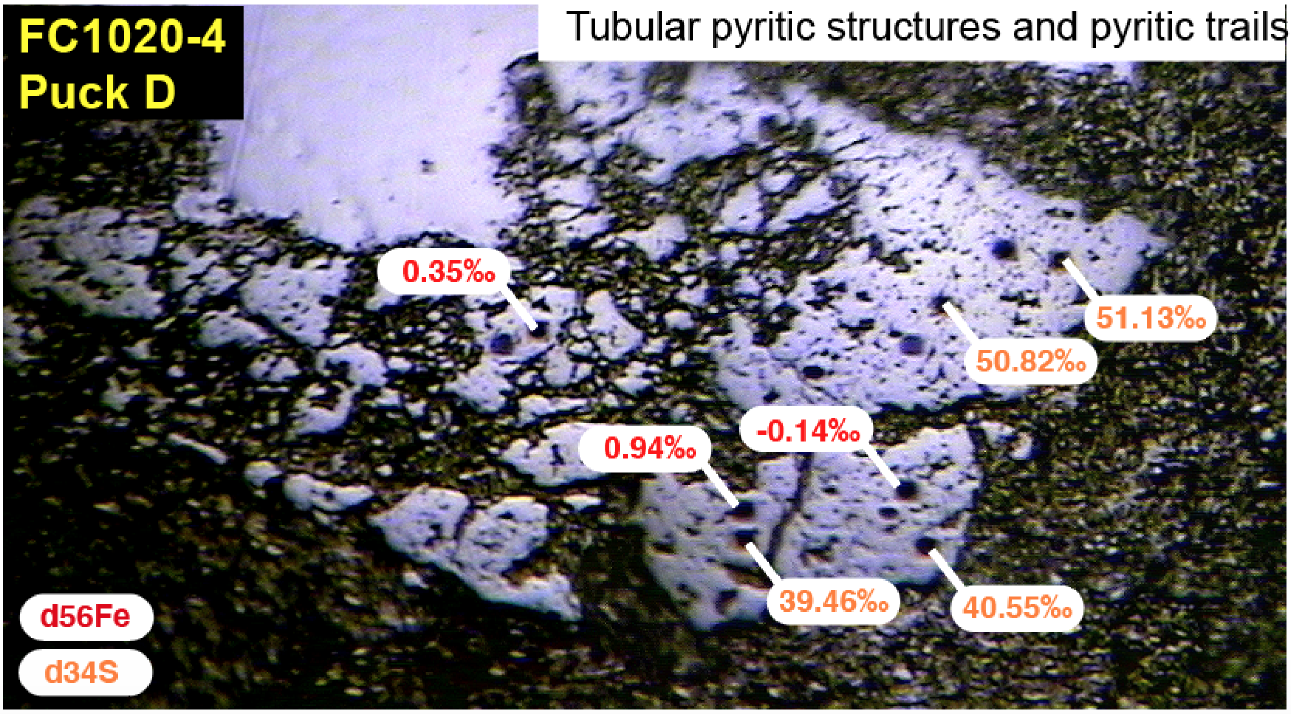
FC1020-4
Puck D
Tubular pyritic structures and pyritic trails
0.35‰
51.13‰
50.82‰
0.94‰
-0.14‰
39.46‰
40.55‰
d56Fe
d34S

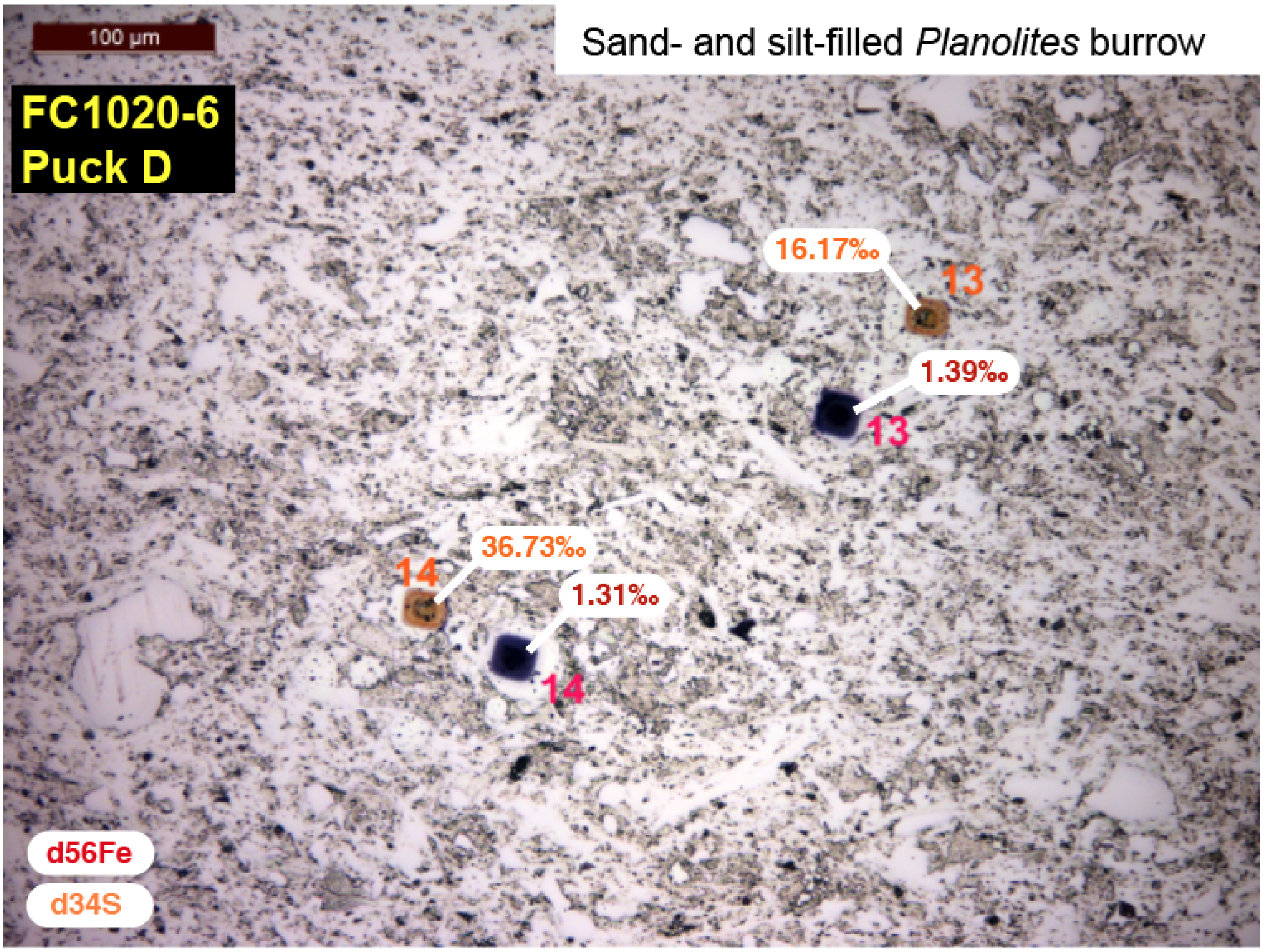
100 µm
Sand- and silt-filled *Planolites* burrow
FC1020-6
Puck D
16.17‰
13
1.39‰
13
36.73‰
14
1.31‰
14
d56Fe
d34S

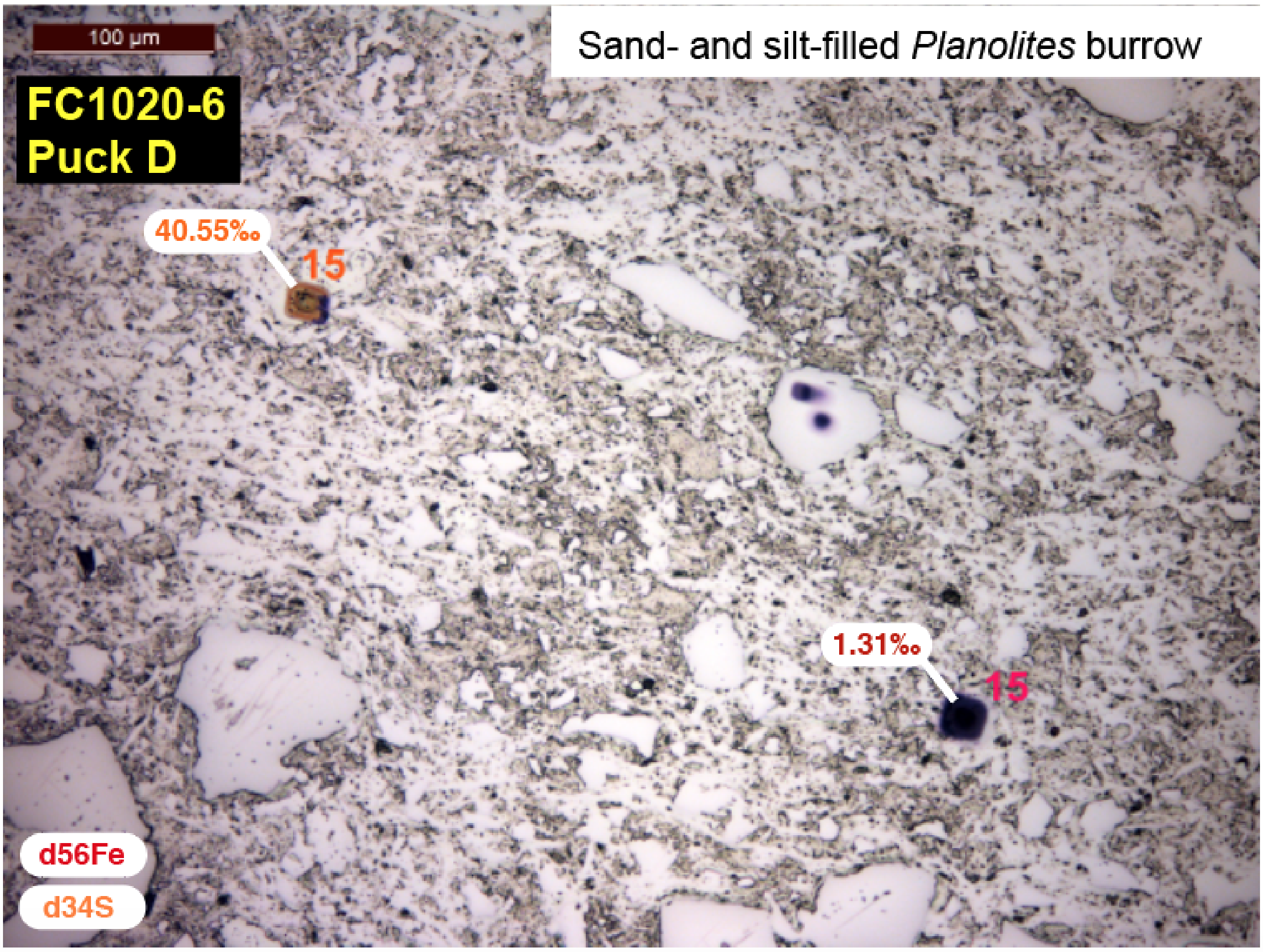
100 µm
Sand- and silt-filled *Planolites* burrow
FC1020-6
Puck D
40.55‰
15
1.31‰
15
d56Fe
d34S

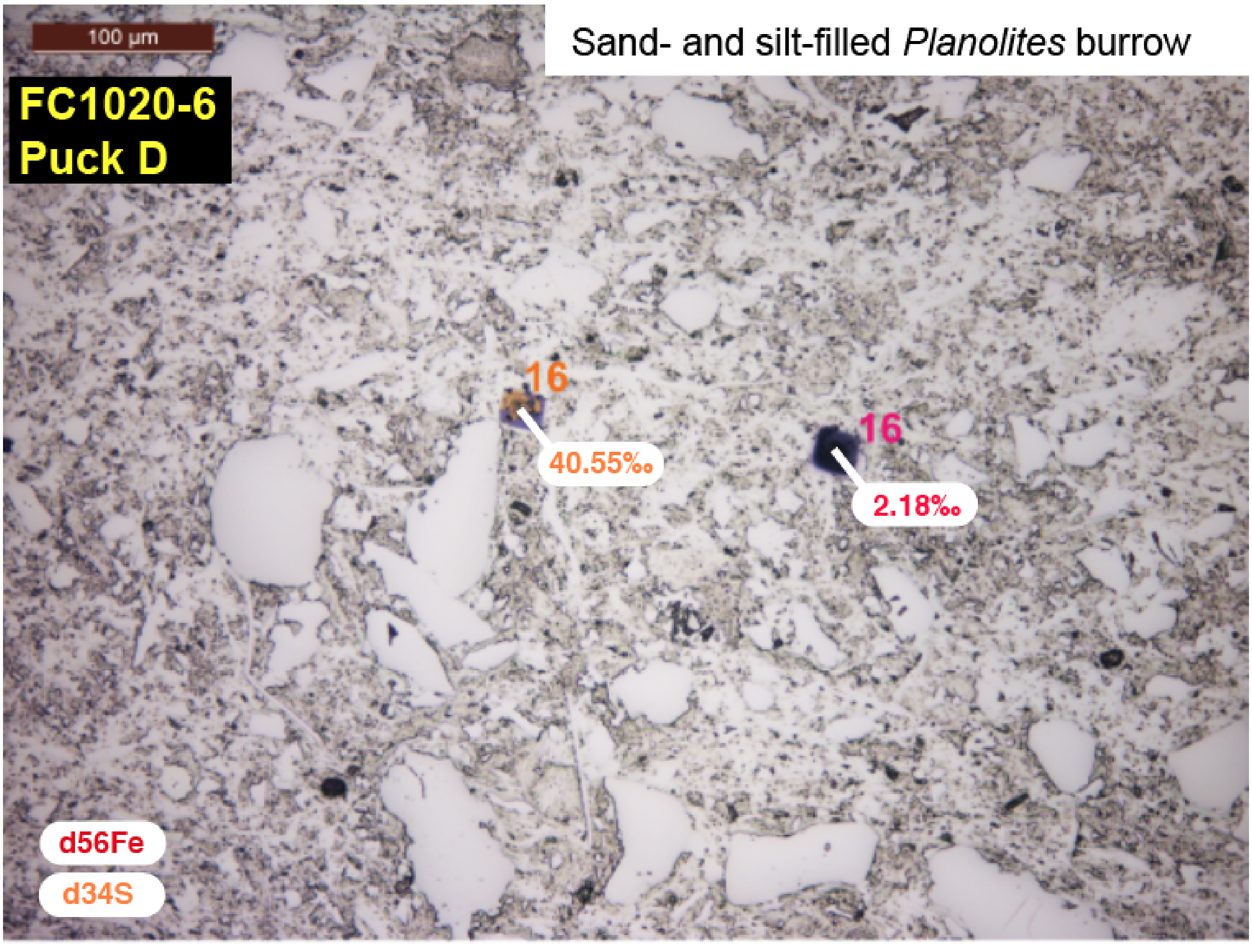

Sand- and silt-filled *Planolites* burrow
100 µm
FC1020-6
Puck D
16
40.55‰
16
2.18‰
d56Fe
d34S


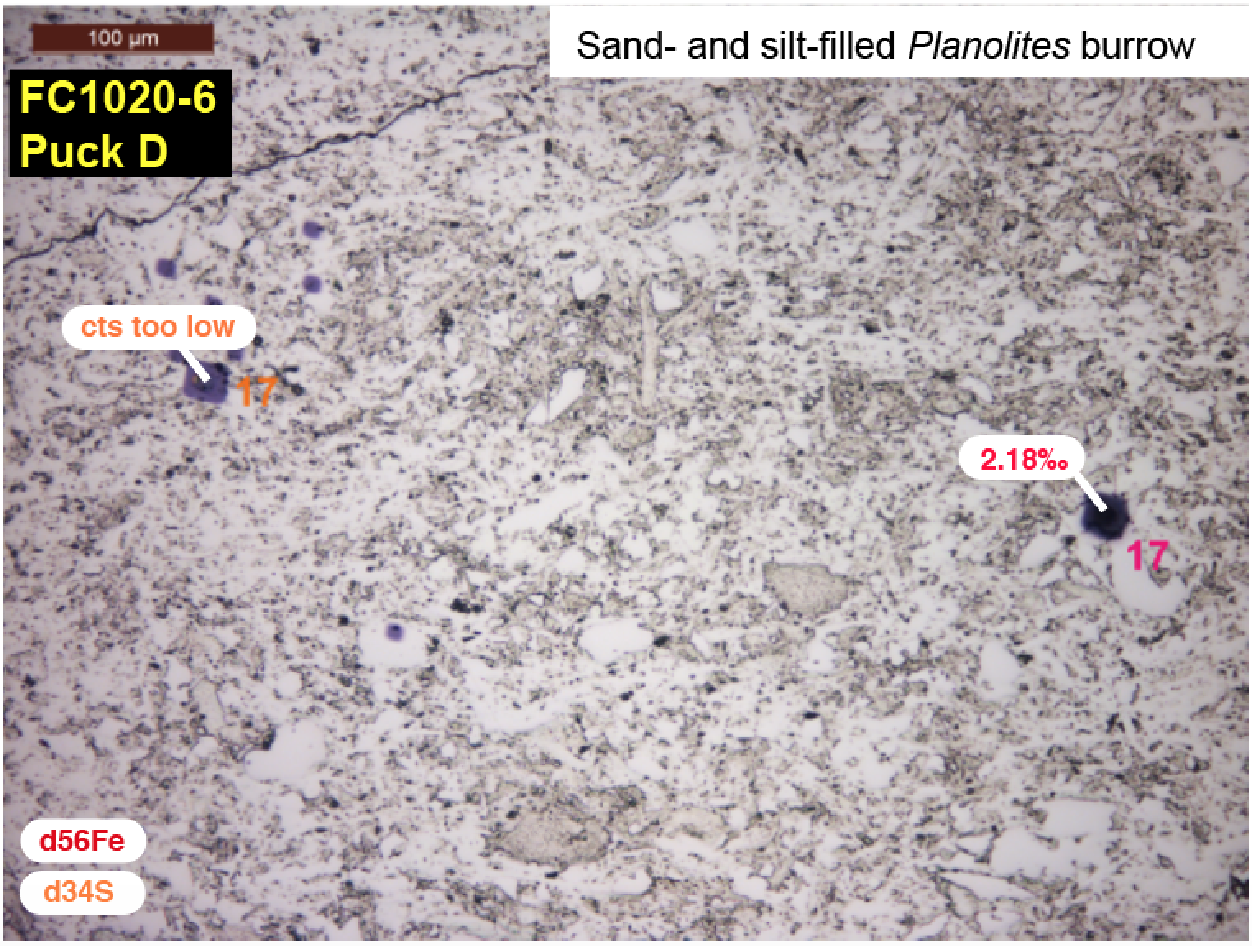

Sand- and silt-filled *Planolites* burrow
100 µm
FC1020-6
Puck D
cts too low
17
2.18‰
17
d56Fe
d34S
